# Genotypic characterization and antimicrobial susceptibility of human *Campylobacter jejuni* isolates in Southern Spain

**DOI:** 10.1128/spectrum.01028-24

**Published:** 2024-08-20

**Authors:** Pablo Fernández-Palacios, Fátima Galán-Sánchez, Carlos S. Casimiro-Soriguer, Estefanía Jurado-Tarifa, Federico Arroyo, María Lara, J. Alberto Chaves, Joaquín Dopazo, Manuel A. Rodríguez-Iglesias

**Affiliations:** 1 UGC Microbiología, Hospital Universitario Puerta del Mar, Cádiz, Spain; 2 Plataforma Andaluza de Medicina Computacional, Fundación Pública Andaluza Progreso y Salud, Sevilla, Spain; 3 Instituto de Investigación e Innovación Biomédica de Cádiz (INIBICA), Hospital Universitario Puerta del Mar, Cádiz, Spain; 4 Subdirección de Protección de la Salud, Consejería de Salud y Familias, Sevilla, Spain; 5 Departamento de Biomedicina, Biotecnología y Salud Pública, Universidad de Cádiz, Cádiz, Spain; Universidad Andres Bello, Santiago, Chile

**Keywords:** *Campylobacter jejuni*, whole-genome sequencing, antimicrobial resistance, virulome, epidemiology

## Abstract

*Campylobacter jejuni* is the main cause of bacterial gastroenteritis and a public health problem worldwide. Little information is available on the genotypic characteristics of human *C. jejuni* in Spain. This study is based on an analysis of the resistome, virulome, and phylogenetic relationship, antibiogram prediction, and antimicrobial susceptibility of 114 human isolates of *C. jejuni* from a tertiary hospital in southern Spain from October 2020 to June 2023. The isolates were sequenced using Illumina technology, and a bioinformatic analysis was subsequently performed. The susceptibility of *C. jejuni* isolates to ciprofloxacin, tetracycline, and erythromycin was also tested. The resistance rates for each antibiotic were 90.3% for ciprofloxacin, 66.7% for tetracycline, and 0.88% for erythromycin. The fluoroquinolone resistance rate obtained is well above the European average (69.1%). CC-21 (*n* = 23), ST-572 (*n* = 13), and ST-6532 (*n* = 13) were the most prevalent clonal complexes (CCs) and sequence types (STs). In the virulome, the *cadF, ciaB*, and *cdtABC* genes were detected in all the isolates. A prevalence of 20.1% was obtained for the genes *wlaN* and *cstIII*, which are related to the pathogenesis of Guillain-Barré syndrome (GBS). The prevalence of the main antimicrobial resistance markers detected were CmeABC (92.1%), RE-cmeABC (7.9%), the T86I substitution in *gyrA* (88.9%), *bla_OXA-61_* (72.6%)*, tet(O)* (65.8%), and *ant (6)-Ia* (17.1%). High antibiogram prediction rates (>97%) were obtained, except for in the case of the erythromycin-resistant phenotype. This study contributes significantly to the knowledge of *C. jejuni* genomics for the prevention, treatment, and control of infections caused by this pathogen.

**IMPORTANCE:**

Despite being the pathogen with the greatest number of gastroenteritis cases worldwide, *Campylobacter jejuni* remains a poorly studied microorganism. A sustained increase in fluoroquinolone resistance in human isolates is a problem when treating *Campylobacter* infections. The development of whole genome sequencing (WGS) techniques has allowed us to better understand the genotypic characteristics of this pathogen and relate them to antibiotic resistance phenotypes. These techniques complement the data obtained from the phenotypic analysis of *C. jejuni* isolates. The zoonotic transmission of *C. jejuni* through the consumption of contaminated poultry supports approaching the study of this pathogen through “One Health” approach. In addition, due to the limited information on the genomic characteristics of *C. jejuni* in Spain, this study provides important data and allows us to compare the results with those obtained in other countries.

## INTRODUCTION

*Campylobacter jejuni* is the main cause of bacterial diarrheal disease worldwide. In 2022, 137,107 cases and 34 deaths associated with campylobacteriosis were reported in Europe. These data remain stable compared with those obtained in 2021 (1). In the United States, surveillance data from the Foodborne Diseases Active Surveillance Network (FoodNet) indicate 9,751 cases of campylobacteriosis in humans and 42 deaths associated with this disease in 2022. According to FoodNet, *Campylobacter* accounts for almost 40% of foodborne infections (2). Although infections are usually self-limiting, *Campylobacter* can also cause severe infections such as bacteremia, sepsis, arthritis, endocarditis, or Guillain–Barré syndrome (GBS) (3). Spain has the highest combined resistance rate to ciprofloxacin and erythromycin (3.1%) among the human isolates of *C. jejuni* in Europe (4).

Point mutations in the *gyrA* gene are the principal mechanism of resistance in *Campylobacter* (5). Several mutations in *gyrA* gene have been described such as T86I, T86K, A70T, and D90N. The C257T change in the *gyrA* gene, leading to the T86I substitution, is the most prevalent mutation among *Campylobacter* isolates (6). In tetracyclines, antibiotic resistance is generated by the presence of the *tet(O*) gene. In macrolides, resistance is caused by mutations in the 23S ribosomal RNA gene and the presence of the *erm(B*) gene, which encodes a methylase. Resistance to beta-lactams in *Campylobacter* is mainly due to the beta-lactamase OXA-61 encoded by the *bla_OXA-61_ gene* (7, 8). Several studies have revealed the presence of genes that encode aminoglycoside-modifying enzymes, such as *the ant (6)-Ia* and *aph(3')-III* genes, in *Campylobacter* (9, 10). In addition, antibiotic efflux pumps (CmeABC, CmeFED, and CmeG) contribute to antibiotic resistance (11). Furthermore, the presence of the resistance-enhancing variant CmeABC (named RE-CmeABC), which significantly increases resistance to ciprofloxacin and erythromycin, has been described in human isolates of *C. jejuni* (12).

The mechanisms underlying the pathogenicity and virulence of *C. jejuni* are not fully understood. However, several genes involved in motility, cell cytotoxicity, invasion, chemotaxis, and the cellular stress response have been described (13, 14). Virulence factors (*wlaN, cgtB, cstII,* and *cstIII*) implicated in the pathogenesis of GBS via the modification of lipooligosaccharide (LOS) have also been studied (15, 16).

Whole-genome sequencing (WGS) has allowed significant advances in the field of molecular epidemiology using the cgMLST (multilocus core genome sequence typing) and wgMLST (whole genome multilocus sequence typing) schemes (17). Both of these approaches have greater discriminatory power than the traditional MLST scheme (18).

To date, there is little information available on the WGS data obtained from *Campylobacter* clinical isolates in Europe (19–21) and Spain. The objective of this study was to analyze the phenotypic and genotypic characteristics of 114 clinical *C. jejuni* isolates from southern Spain. Advanced WGS tools enable a more in-depth understanding of the resistome, virulome, and epidemiology. In this way, we can better understand the pathogenicity and nature of *C. jejuni*, a highly important zoonotic pathogen for public health.

## MATERIALS AND METHODS

### Isolates

A total of 114 *C*. *jejuni* isolates, recovered from unformed stool from patients with diarrhea submitted to the microbiology laboratory of a tertiary hospital in southern Spain from October 2020 to June 2023, were included in the study. The patients came from the cities of Cadiz and San Fernando. One hundred twenty-six isolates were recovered during the study period, but 12 were not included in the analysis due to the low quality of the sequences obtained by WGS. The samples were cultured in blood-free BD Campylobacter selective medium (Beckton Dickinson, New Jersey, USA) and incubated at 42°C for 48 hours under microaerobic conditions (85% nitrogen, 10% carbon dioxide, and 5% oxygen). The *C. jejuni* isolates were confirmed by mass spectrometry using MALDI-TOF (Bruker, Bremen, Germany).

### Antimicrobial susceptibility testing

Antimicrobial susceptibility testing was performed using the disk diffusion method according to the European Committee on Antimicrobial Susceptibility Testing (EUCAST) recommendations (22). The antibiotics tested included ciprofloxacin (5 µg), erythromycin (15 µg), and tetracycline (30 µg) (Oxoid, Basingstoke, UK). The isolates were incubated for 24–48 hours under microaerophilic conditions, and the zones of antibiotic inhibition were interpreted according to EUCAST breakpoints 2023 (22).

### Whole-genome sequencing and data analysis

Bacterial DNA was extracted from *C. jejuni* isolates using an EZ1 Advanced XL (Qiagen, Hilden, Germany) following the manufacturer’s instructions. The quantity of extracted bacterial DNA was measured using a Qubit 3.0 fluorometer (Fisher Scientific, Massachusetts, USA) and a Qubit dsDNA HS assay kit. Libraries were prepared using the Nextera XT DNA Library Preparation Kit (Illumina, Cambridge, UK) according to the manufacturer’s instructions and quantified using the Qubit 3.0 fluorometer. MiSeq (Illumina, Cambridge, UK) was used for the genome sequencing of the isolates. Quast (23), Kraken (24), Fastp (25), Qualimap (26), and Checkm (27) were used to evaluate the quality of the FASTQ files obtained in the sequencing process and the FASTA files of the assembled genomes. *De novo* assembly was performed using SPAdes (28). The data obtained from the use of these bioinformatics tools and those obtained through the analysis of *C. jejuni* sequences were deposited in the SIEGA app (Andalusia Integrated Molecular Epidemiology System) (29). The genome metrics of the isolates are available in Table S1 in the supplemental material.

### Clonal complex (CC), multilocus sequence typing (MLST), and core genome MLST (cgMLST) analyses

The MentaLiST application (30) was used to obtain allele profiles from *C. jejuni* isolates. Based on these allelic profiles, the sequence types (STs) and CCs of the isolates were determined using the MLST scheme, which is based on the variation in seven highly conserved genes (*aspA, glnA, gltA, glyA, pgM, tkt,* and *uncA*). Core genome MLST (cgMLST) analysis of the isolates was conducted using allelic profiles based on 1,343 core genome genes of *Campylobacte*r (31) sourced from the PubMLST database. The *C. jejuni*/*C. coli* cgMLST v1 version, updated as of January 5, 2024, was obtained from PubMLST. The alleles were identified using an in-house developed script. A neighbor-joining phylogenetic tree was constructed to establish the phylogenetic relationships among the isolates based on allelic distances from the cgMLST scheme. The phylogenetic results and annotations were visualized using iTOL v.6 (32)

### Identification of virulence factors

The Virulence Factor Database (VFDB) (33) was used to determine the virulence factors present in the *C. jejuni* isolates. From the total virulence genes obtained by VFBD, *cadF* (cell adhesion), *ciaB* (invasion), and *cdtABC* (cytotoxin production) were analyzed. The presence of genes related to GBS pathogenesis that modify the structure of lipopolysaccharides (LOS), such as *wlaN* and *cstIII*, and genes related to the type IV secretion system (T4SS), such as *VirB* genes, was also investigated. VFDB analysis search for the T4SS associated with pVir plasmid.

### Identification of antimicrobial resistance markers and correlation between phenotype and genotype

The antimicrobial resistance markers (*tet(O*) gene, *erm(B*) gene*,* aminoglycoside resistance genes, blaOXA*_-61_* gene, and *cmeABC* operon) present in the *C. jejuni* sequences were obtained using the ABRicate pipeline (34). The databases used were Resfinder (35), CARD (36), MEGARes (37), and ARG-ANNOT (38). Chromosomal mutations in the *gyrA* and 23S rRNA genes were obtained using PointFinder (39), which is included in Resfinder version 4.4.2. Correlations between the resistance phenotypes to ciprofloxacin, erythromycin, and tetracycline and the genotypes (antimicrobial resistance markers) of 114 *C*. *jejuni* isolates were determined. The blastn tool (40) was used to search for RE-cmeABC variant in the isolates by comparing the genome of RE-cmeABC and the cmeB subunit of the reference strain *Campylobacter jejuni* subsp. *jejuni* NCTC 11168.

## RESULTS

### Phenotypic results of antimicrobial susceptibility testing

Antibiotic susceptibility tests were performed on 114 isolates of *C. jejuni* (see Table S2 in the supplemental material). All results were interpreted at 24 hours according to EUCAST breakpoints. The resistance rates for each antibiotic were 90.3% for ciprofloxacin, 66.7% for tetracycline, and 0.88% for erythromycin. Twenty-eight isolates (24.6%) were resistant to at least one antibiotic, whereas 77 isolates (67.5%) were resistant to at least two antibiotics. Ciprofloxacin and tetracycline resistance (65%) was the most common phenotype among the *C. jejuni* isolates. Nine isolates (7.9%) were susceptible to the three antibiotics. Only one isolate (cjeju22_21) was resistant to erythromycin. Table 1 shows the antibiotic resistance data and the number of antibiotics used for each observed phenotype.

**TABLE 1:** Antimicrobial susceptibility testing performed on 114 isolates of *C. jejuni^a^*

Number of resistant antimicrobial groups	Phenotype (CIP, TET, and E)	Number of isolates (%)
0	(S,S,S)	9 (7.9)
1	(R,S,S)	28 (24.6)
1	(S,R,S)	2 (1, 7)
2	(R,R,S)	74 (64.9)
2	(R,S,R)	1 (0.9)

^*a*^ The antibiotics tested were ciprofloxacin (CIP), tetracycline (TET), and erythromycin (E).

### Phylogenetic study of *C. jejuni* isolates

**Fig 1:**
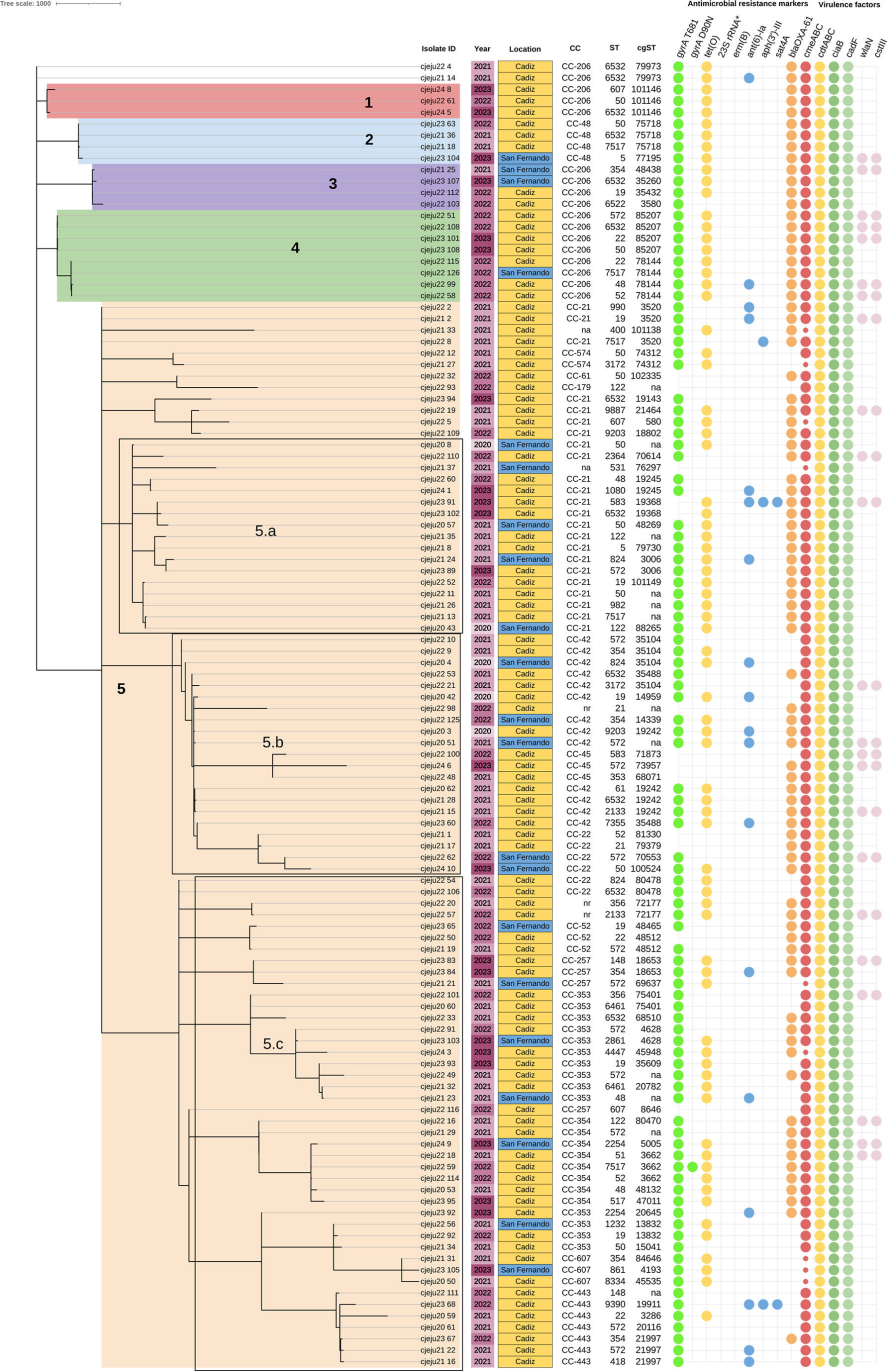
Phylogenetic analysis of 114 isolates of *C. jejuni*. A neighbor-joining phylogenetic tree was constructed to establish the phylogenetic relationships among the isolates based on allelic distances from the cgMLST scheme. The clades are denoted by the numbers 1, 2, 3, 4, and 5. Clade 5 is divided into subclades 5.1, 5.2, and 5.3. Clonal complexes (CCs), sequence types (STs), and core genome sequence types (cgSTs) are listed on the labels of each isolate. The presence of antimicrobial resistance at markers and virulence factors is visualized by circles in colors for each gene. Location (Cadiz and San Fernando) and year of isolation (from 2021 to 2023) are represented in colors. The isolates that could not be assigned a single cgST are labeled “na” (not assigned). The presence of the *RE-cmeABC* operon is indicated by a small red circle. (*) 23S rRNA mutations.

A variety of 14 CCs, 41 STs, and 68 different cgSTs were obtained among the 114 isolates of *C. jejuni* (see Table S3 in the supplemental material). CC-21 (*n* = 23), CC-206 (*n* = 17), and CC-353 (*n* = 14) were the most prevalent CCs. ST-572 (*n* = 13), ST-6532 (*n* = 13), and ST-50 (*n* = 10) were the most prevalent STs. Among the wide variety of cgTs detected, the most prevalent was cg85207 (four isolates distributed across two clusters of two cases each between 2022 and 2023), cg78144 (four isolates distributed across two clusters of two cases each in 2022), cg35104 (four isolates distributed across two clusters of two cases each between 2020 and 2021), and cg19242 (four isolates distributed across two clusters of two cases each between 2020 and 2021) (Fig. 1).

Five isolates (4.4%) did not belong to the described CCs but were included in 4 STs: ST-2133 (*n* = 2), ST-2861 (*n* = 1), ST-1080 (*n* = 1), and ST-531 (*n* = 1). For 12 isolates, an exact cgMLST profile could not be assigned; more than one cgMLST profile was detected according to the *C. jejuni/C. coli* cgMLST scheme (see Table S3 in the supplemental material). The CCs, STs, and cgSTs of each isolate are shown in Fig. 1.

Phylogenetic analysis revealed that the *C. jejuni* isolates clustered into five different clades. Two isolates (cjeju22_4 and cjeju21_14) did not cluster into any clade (Fig. 1). Clade 1 was composed of three isolates recovered in Cadiz in the same year and shares the same CC (CC-206) and cgST (cg101146), although they were distributed in three STs (Fig. 1). Clade 2 and clade 3 were each composed of four isolates. Isolates belonging to these clades were recovered in Cadiz and San Fernando between 2021 and 2023 and share the same CC among them, CC-206 for the isolates of clade 2 and CC-48 for clade 3. However, isolates belonging to clade 2 and clade 3 were grouped into different STs and cgSTs (Fig. 1). Clade 4 was composed of eight isolates, seven of which were recovered from Cadiz and one from San Fernando between 2022 and 2023. All isolates in this clade belonged to CC-206, and they were clustered into seven STs and two cgSTs (cg85207 and cg78144) (Fig. 1). Clade 5 comprised 93 isolates, which were further divided into three subclades (5.1, 5.2, and 5.3). Of the 93 isolates belonging to clade 5, 12 did not belong to any subclade (Fig. 1). The number of clade 5 isolates varied greatly according to the year of isolation, geographical distribution, and epidemiology. None of the five clades showed a clear distribution of any specific antimicrobial resistance marker.

### Virulence factors

A wide variety of virulence factors were detected in the *C. jejuni* isolates (Fig. 1). The most prevalent virulence factors were associated with cell adhesion (*cadF*), invasion (*ciaB*), and cytotoxin production (*cdtABC*) and were detected in all the *C. jejuni isolates*. The *wlaN* and *cstIII* genes, which are implicated in the pathogenesis of GBS, were detected in 23 isolates (20.15%). These 23 isolates were distributed in seven CCs (Fig. 1). The *wlaN* and *cstIII* genes were detected mainly in CC-21 (*n* = 13) and CC-206 (*n* = 4). Three out of four isolates belonged to the cg85207 group, which carries the *wlaN* and *cstIII* genes (Fig. 1). Genes related to the type 4 secretion system (T4SS) were not detected in any isolate of *C. jejuni*. The virulence factors details of the isolates are available in Table S1 in the supplemental material. All virulence factors detected in the 114 *C*. *jejuni* isolates are shown in Table S6 of the supplemental material.

### Antimicrobial resistance markers

*GyrA* mutations were detected in 101 isolates (88.6%). The T86I substitution was detected in these 101 isolates. In addition, the D90N substitution was detected in isolate cjeju22_59. The *tet(O*) gene was identified in 75 isolates (65.8%). Regarding aminoglycoside resistance genes, the *ant (6)-Ia* gene was detected in 19 isolates (16.7%). In the isolates cjeju23_68 and cjeju23_91, the *ant (6)-Ia* gene, *aph(3')-III* gene, and *sat-4* gene were detected (Fig. 1). The *aph(3')-III* gene encodes a phosphotransferase that confers resistance to kanamycin, amikacin, and other aminoglycosides. The *sat-4* gene encodes resistance to streptomycin. Furthermore, *the aph(3')-III* gene was detected in the isolate cjeju22_8 (Fig. 1).

A high distribution of the *cmeABC* operon was detected among the 105 *C*. *jejuni* isolates (92.1%). In nine isolates (7.9%), the *cmeA* and *cmeC* subunits, but not the *cmeB* subunit, were detected (Fig. 1). In these nine isolates, the presence of RE-CmeABC was detected (95% identity) using the blastn tool, leading to the hypothesis that these isolates carry RE-CmeABC. The *bla_OXA-61_* gene was detected in 83 isolates (72.8%). Point mutations in the 23S rRNA and *erm(B*) genes were not detected in any of the isolates. All resistance markers detected in the *C. jejuni* isolates are shown in Fig. 1.

### Correlation between resistance phenotype and antimicrobial resistance markers

Correlations were determined between antimicrobial susceptibility test (phenotype) and WGS (genotype) data (see Table S4 in the supplemental material). The substitution of T68I in the *gyrA* gene was detected in 102 isolates resistant to ciprofloxacin but was not detected in any of the isolates susceptible to ciprofloxacin, with correlations of 98.1% and 100%, respectively. Seventy-five of the 76 isolates resistant to tetracycline carried the *tet(O*) gene, indicating a correlation between the phenotype and genotype of 98.7%. Similarly, none of the isolates susceptible to tetracycline carried the *tet(O*) gene, showing a 100% correlation. Resistance markers for erythromycin resistance were not detected among the susceptible isolates, indicating a 100% correlation between phenotype and genotype. One isolate was resistant to erythromycin, but neither the *erm(B*) gene nor any mutations in 23S rRNA were detected. The minimum inhibitory concentration (MIC) of ciprofloxacin was determined for eight of the nine isolates in which the RE-cmeABC variant was detected. An MIC >32 mg/L was obtained for seven of the eight tested isolates, and an MIC of 0.125 mg/L was obtained for the cjeju21_37 isolate, which was categorized as susceptible according to the EUCAST breakpoints. The correlations between the phenotypes and genotypes of *C. jejuni* isolates treated with ciprofloxacin, tetracycline, and erythromycin are presented in Table 2.

**TABLE 2:** Correlation between the phenotype and genotype of the 114 isolates of *C. jejuni^a^*

Antibiotic	Number of isolates R or S	Number of isolates carrying resistance markers	Rate of correlation between phenotype/genotype (%)
Ciprofloxacin	R: 103	100 (*gyrA* T86I) 1 (*gyrA* T86I *+ gyrA* D90N)	98.1 (101/103)
	S: 11	0	100 (11/11)
Erythromycin	R: 1	0	0
	S: 113	0	100 (113/113)
Tetracycline	R: 76	75 (*tet(O*))	98.7 (75/76)
	S: 38	1 (*tet(O*))	97.4 (37/38)

^*a*^ Antimicrobial resistance markers detected for each clinical category for ciprofloxacin, tetracycline, and erythromycin are shown. The isolates were classified by clinical category according to the EUCAST breakpoints: resistant (R) and susceptible standard dosing (S) regimens.

## DISCUSSION

This analysis, performed on clinical isolates of *C. jejuni,* included the study of the resistome and virulome and phylogenetic analysis using whole-genome sequencing, as well as phenotypic antimicrobial susceptibility data. To date, few genomic studies on the genomic characteristics of human *C. jejuni* isolates from Spain have been published.

Ciprofloxacin, doxycycline, and azithromycin are the antibiotics most commonly used for the treatment of *Campylobacter*-related gastroenteritis. Due to its low resistance rate, azithromycin and erythromycin are considered the antibiotics of choice (41). The resistance rates for ciprofloxacin and tetracycline determined in this study (90.3% and 66.7%, respectively) are higher than the European averages of 69.1% and 46.6%, respectively (4), but are similar to those reported for Spanish isolates of *C. jejuni* in 2022 (42). The ciprofloxacin resistance observed in the isolates from this study is higher than those observed in other similar studies in the rest of the world. In Europe, studies in Italy and Poland showed ciprofloxacin resistance rates of 72.1% and 74.4%, respectively (19, 43). On other continents, resistance rates range from 20%−30% according to data provided by studies on *C. jejuni* isolates from Australia, the USA, and South America (44–46). Within South America, Peru has one of the highest ciprofloxacin resistance (84.72%). In a study conducted in Taiwan, the ciprofloxacin resistance rate (91.1%) in human *C. jejuni* isolates does resemble that obtained in this study (47). Despite the very worrying data on fluoroquinolone resistance in some countries, *C. jejuni* is no longer part of the WHO list of priority pathogens after the last update in 2024 (48). In light of the data obtained in this study, the status of *C. jejuni* according to the WHO could be reconsidered in the future.

Phylogenetic analysis revealed that CC-21 is the most common clonal complex in our area (20.2%). This finding is consistent with genomic studies conducted in South America (49–51) and Europe (19–21). In a study that included 108 Peruvian isolates of *C. jejuni,* CC-362 was the most prevalent clonal complex, followed by CC-21 (51). In addition to CC-21, Spanish isolates were grouped mainly into CC-206 (11.4%) and CC-353 (11.4%), with significantly greater percentages than those of PubMLST (5.3% and 5.5%, respectively) (52).

Among the varieties of STs obtained, 10 (8.8%) were ST-50. ST-50 is widely distributed around the world and is detected in greater or lesser proportions depending on the geographical area (19–21, 49). In this study, the most common STs were ST-572 (11.4%) and ST-6532 (11.4%). According to the data of clinical isolates uploaded to PubMLST (52), two of the 103 Spanish isolates were ST-572, and none were ST-6532. ST-572 has been detected almost entirely in Europe in animal and human isolates. According to PubMLST (52), ST-6532 is less widely distributed than ST-572 and is detected in 15 isolates, most of which are in Great Britain. The results of this analysis of isolates from southern Spain are partially in agreement with those obtained in other European countries with respect to the distribution of ST-572 and ST-50 (52). However, in this geographic area, a greater distribution of ST-6352 was detected among the Spanish isolates. ST-6532 was detected in three isolates from a genomic study of 40 *C*. *jejuni* isolates from ruminants in Vizcaya (Northern Spain) (53). In a study of isolates from various sources in Spain, any human isolates of *C. jejuni* were grouped with ST-6532 (54).

*C. jejuni* can be isolated from several sources, including environmental, animal, and human sources. Therefore, there is a possibility of a “one-health” approach to the study of this microorganism. Several clonal complexes have been isolated from several sources and are known as CC “host generalists” (CC-21, CC-48, CC-206, and CC-45) (54). In this study, a greater percentage of *C. jejuni* isolates belonging to the CC-21 complex (20.2%) and ST-206 complex (14.9%) was obtained. The prevalence of the ST-48 and ST-45 complexes was much lower, at 3.5 and 2.6, respectively. According to PubMLST, 18.2% of *C. jejuni* isolates from chickens belong to CC-353, which is the most prevalent CC in this animal. Although it is considered a chicken-specific CC, cases of Campylobacteriosis in humans belonging to this CC have been described (5.5% of *C. jejuni* isolates from human feces according to PubMLST) (52). In the area studied in this study, a higher prevalence of CC-353 (12.3%) was detected, which highlights the zoonotic transmission of *C. jejuni*. The isolates belonging to CC-353 were grouped in subclade 5.3, distributed in the two areas studied and recovered between 2021 and 2023 (Fig. 1). In addition, this study included isolates belonging to chicken-specific CCs, such as ST-354 and ST-443, although they were distributed in a lower proportion than CC-353 (6.1% for both CCs).

Associations between some STs of *C. jejuni* isolates and antibiotic resistance have been observed. Increasing fluoroquinolone resistance among human *C. jejuni* isolates is a public health concern. In this regard, the rate of resistance to fluoroquinolones among *C. jejuni* isolates belonging to ST-50 is approximately 60%, according to studies carried out in Brazil, Belgium, and Germany (50, 55, 56). For ST-572, in the Belgian research, a fluoroquinolone resistance of 80% was obtained among *C. jejuni* isolates belonging to this ST (55). Furthermore, in a study of more than 300 *C*. *jejuni* isolates in Switzerland, ST-572 and ST-19 were the STs with the highest resistance to ciprofloxacin (57). Thus, several studies point to an association between ciprofloxacin resistance and ST-50. Of the few published data on ST-6532, all isolates belonging to this clone in northern Spain were resistant to ciprofloxacin (53). In our study, all isolates of *C. jejuni* belonging to ST-50, ST-6532, and ST-572 were resistant to ciprofloxacin, showing greater resistance than that reported in other published studies. In addition to the relationship between antibiotic resistance and known STs of *C. jejuni*, new STs belonging to multiresistant *C. jejuni* isolates have been described in recent years. ST-6964 in New Zealand (58) and ST-5136 in the UK (59) are two examples.

Analysis of the cgMLST profiles of the isolates revealed a heterogeneous distribution without a clear predominance of any specific cgST. Cg35104, cg19242, cg85207, and cg78144 were the most prevalent, although not in a large proportion of the total isolates. The isolates in our study were grouped into five different clades with high genetic diversity. In the study of phylogenetic clades, previous studies in Peru and Poland have shown that associations between clusters and the presence of antimicrobial resistance markers and virulence factors are common (51, 60). In our study, we did not observe an epidemiological relationship in this regard. Furthermore, there was no observable distribution based on geographic area, Cadiz or San Fernando (Fig. 1), as these populations are very close, and there was a significant interaction between the populations of both cities.

The mechanisms of antibiotic resistance in *Campylobacter* have been widely studied. However, the pathogenic mechanisms and the mode of interaction of *Campylobacter* with the host are not fully defined (12, 61). A prevalence of 21% was detected for the *wlaN* and *cstIII* genes, with a possible association with ST-50 because these two genes were detected in 80% of the isolates belonging to ST-50 (Fig. 1). The prevalence of the *wlaN* gene is similar to that obtained in a study conducted in northern Spain, where the *wlaN* gene was detected in 20% of human isolates. However, there is no clear predominance of any ST that could indicate a relationship between ST and the distribution of this gene (15).

The C257T mutation, which causes the substitution of T68I in the *gyrA* gene, is the main mechanism of resistance to ciprofloxacin reported in several studies (19, 20, 49, 50). In our study, the T68I substitution was detected in almost 90% of the isolates (Fig. 1). Other mutations in the *gyrA* gene that also confer resistance to ciprofloxacin have been described, such as D90N and T86K, although they are detected in smaller proportions (6). In the isolate cjeju22_59, the D90N substitution was detected along with the T86I substitution. The percentage of detection of the *tet(O*) gene, *bla_OXA-61_*, and *cmeABC* operon aligns with the findings of previous studies conducted in other countries (20, 49, 50, 54). The main gene encoding aminoglycoside resistance genes was *ant (6)-Ia* (16.7%), which confers resistance to streptomycin. This gene was detected in the same proportion in studies performed in North America and South America in *C. jejuni* clinical isolates (62). The *aph(3')-III* gene was detected in three isolates (cjeju23_92, cjeju22_49, and cjeju24_3). In isolates cjeju22_49 and cjeju24_3, *t*he *aph(3’)-III* gene was detected along with the *ant (6)-Ia* gene and the *sat-4* gene. These three genes can be included in these two isolates, forming the (*ant (6)-sat4-aph(3′)-III*) cluster. This cluster has been detected in the chromosomal and plasmid genomes of gram-positive bacteria such as *Streptococcus* (63). No antimicrobial resistance markers for erythromycin or type IV secretion (T4SS) were detected, which is consistent with the low prevalence of these genes in clinical isolates of *C. jejuni* (20, 49, 64).

Another interesting result of our study is the detection of the RE-CmeABC variant in human *C. jejuni* isolates in Spain. Antibiotic resistance databases do not include the sequence of this efflux pump, and there are few published studies to date about the characteristics of RE-CmeABC, resulting in a lack of information about this resistance mechanism in *C. jejuni*. The presence of this RE-CmeABC variant can be suspected when the *cmeB* subunit from the CARD (65) is not detected, and atypical *cmeB* can be detected by comparing the *cmeB* subunit of *C. jejuni* NCTC 1168 with that of an isolate (12). Furthermore, an extremely high MIC of ciprofloxacin (>32 mg/l) was obtained for 87.5% of the isolates where the RE-cmeABC variant was detected. Although ciprofloxacin MICs are not available for all the isolates included in this study, these MIC data suggest that the RE-cmeABC variant contributes to increasing antibiotic resistance to fluoroquinolones. In the seven isolates in which the RE-cmeABC variant was detected, the T86I mutation in the *gyrA* gene was also detected in each of the isolates. The isolate cjeju21_37, where the RE-cmeABC variant was detected, has no mutations in the *gyrA* gene and is susceptible to ciprofloxacin. The results for this isolate revealed that the CmeABC efflux pump and the RE-cmeABC variant contribute to increased resistance to fluoroquinolones, but in the absence of mutations in gyrA, it fails to cause antibiotic resistance. Most published studies on the CmeABC efflux pump indicate a synergistic mechanism of action along with other antibiotic resistance mechanisms for fluoroquinolones, macrolides, and tetracyclines (66, 67). However, further studies on the ability of the cmeABC efflux pump to produce antibiotic resistance without an additional resistance mechanism are needed. A recent study conducted in Peru on 97 clinical *C. jejuni* isolates indicated that the prevalence of RE-CmeABC was 36.1% (65). In our study, this variant was detected in 8% of the isolates, 5 of which belonged to subclade 5.3. Three of these isolates exhibited a phylogenetic relationship. These three isolates belong to CC-607, but no epidemiological relationship was detected according to the geographic area or year of isolation (Fig. 1).

WGS-based antibiotic resistance tools have yielded excellent results in predicting antibiogram outcomes. A correlation of more than 98% was obtained between antimicrobial resistance markers and antimicrobial susceptibility tests for ciprofloxacin, tetracycline, and erythromycin among more than 500 isolates of *C. jejuni* in Denmark (68). Other analyses performed in Israel revealed a 96%−100% correlation among 263 isolates of *C. jejuni* (69). These results are similar to those obtained in our analysis of 114 isolates, as a greater than 97% correlation was observed (except for the erythromycin-resistant phenotype). According to these previous studies, tetracycline is the antibiotic with the lowest correlation percentage (68, 69). In this study, *C. jejuni* isolates resistant to ciprofloxacin (two isolates), tetracycline (one isolate), and erythromycin (one isolate) were obtained, and no associated resistance mechanisms were detected (Table 2). Other resistance mechanisms, such as the overexpression of the CmeABC efflux pump, can explain why antimicrobial resistance genes related to these antibiotics were detected in isolates with phenotypic resistance (66, 70, 71)

A limitation of this analysis is that the collection of *C. jejuni* isolates was not performed through a continuous surveillance program but rather from a specific period. This approach may not accurately represent the characteristics of *C. jejuni* in our region. Furthermore, 4.4% of the isolates could not be typed by CC, and 10.5% of the isolates could not be typed by cgMLST, resulting in a loss of relevant information for understanding the local epidemiology of isolates. The VFDB database contains information on the virulence factors encoded by the T4SS associated with the pVir plasmid. The T4SS has been described in other plasmids, such as pTet and pCC31 (53, 72); hence, a more in-depth analysis of the distribution of the T4SS in plasmids hosted in the genomes of *C. jejuni* isolates is needed. Another limitation of this study is that the isolates were recovered from a small geographical area in southern Spain.

In conclusion, our study provides new data about the genotypic and phenotypic characteristics of human *C. jejuni* isolates from Spain. Although *Campylobacter* does not belong to the WHO list of priority public health pathogens, the extremely high rates of fluoroquinolone resistance observed in this study are cause for concern. The results obtained in this work have been deposited in the database of the Integrated Genomic Surveillance System of Andalusia (SIEGA). The SIEGA project was created with the objective of obtaining, through WGS, a genomic database of the most important pathogens for public health in Andalusia. The findings obtained in this work should be correlated with data on animal and environmental isolates, providing data about the relationship.

## Data Availability

The sequences of all the isolates sequenced in this study are available at GenBank (project number PRJNA1088320, https://dataview.ncbi.nlm.nih.gov/?archive=bioproject). The metadata of the isolates and MLST profiles are available at PubMLST (https://pubmlst.org/bigsdb?db=pubmlst_campylobacter_isolates). The sequences used for the analysis of RE-CmeABC were: RE-CmeABC (GenBank genome accession: KT778507.1) and the cmeB subunit (GenBank genome accession: NC_002163.1). Full data on antimicrobial resistance markers, virulence factors, antimicrobial susceptibility testing, and genetic diversity are available in the supplementary files.

## SUPPLEMENTAL MATERIAL

Supplemental material spectrum.01028-24-s0001.docx Tables S1 to S5.

Table S6 spectrum.01028-24-s0002.csv Virulence factors detected in the 114 *C. jejuni* isolates.

ASM does not own the copyrights to Supplemental Material that may be linked to, or accessed through, an article. The authors have granted ASM a non-exclusive, world-wide license to publish the Supplemental Material files. Please contact the corresponding author directly for reuse.
