# Supplementary material for: Genotypic characterization and antimicrobial susceptibility of human Campylobacter jejuni isolates in Southern Spain
Source: Microbiol Spectr. 2024 Aug 20;12(10):e01028-24. doi: 10.1128/spectrum.01028-24 (PMC11449230; doi:10.1128/spectrum.01028-24)
Supplement: Supplemental material — Tables S1 to S5. [file spectrum.01028-24-s0001.docx]

Genotypic Characterization and Antimicrobial Susceptibility of Human *Campylobacter jejuni* Isolates in Southern Spain

Pablo Fernández-Palacios ^a^, Fátima Galán-Sánchez ^a^#, Carlos S Casimiro-Soriguer ^b^, Estefanía Jurado-Tarifa ^c^, Federico Arroyo ^a^, María Lara ^b^, J. Alberto Chaves ^d^, Joaquín Dopazo ^b^, Manuel A. Rodríguez-Iglesias ^a,e^

^a^UGC Microbiología, Hospital Universitario Puerta del Mar, Cádiz, Spain

^b^Plataforma Andaluza de Medicina Computacional, Fundación Pública Andaluza Progreso y Salud, Sevilla, Spain

^c^Instituto de Investigación e Innovación Biomédica de Cádiz (INIBICA), Hospital Universitario Puerta del Mar, Cádiz, Spain

**^d^Subdirección de Protección de la Salud, Consejería de Salud y Familias**, Sevilla, Spain

^e^Departamento de Biomedicina, Biotecnología y Salud Pública, Universidad de Cádiz, Spain

#Address correspondence to Fátima Galán-Sánchez, fatima.galan@uca.es

# Supplemental Tables

**Table** **S1** Genome metrics and accession numbers of 114 *Campylobacter jejuni* isolates.

| **SRA accession** | **Genome accession** | **Biosample_accession** | **Isolate ID** | **Genome coverage** | **N50** | **Number of reads** | **Number of contigs** |
| --- | --- | --- | --- | --- | --- | --- | --- |
| [SRR28623923](https://dataview.ncbi.nlm.nih.gov/object/SRR28623923) | JBBFGA000000000 | SAMN40466906 | cjeju20_3 | 99.98x | 139291 | 5734618 | 40 |
| [SRR28623922](https://dataview.ncbi.nlm.nih.gov/object/SRR28623922) | [JBBFFZ000000000](https://www.ncbi.nlm.nih.gov/nuccore/JBBFFZ000000000) | SAMN40466907 | cjeju20_4 | 99.99x | 179732 | 7119546 | 77 |
| [SRR28623900](https://dataview.ncbi.nlm.nih.gov/object/SRR28623900) | [JBBFFY000000000](https://www.ncbi.nlm.nih.gov/nuccore/JBBFFY000000000) | SAMN40466908 | cjeju20_42 | 99.99x | 159977 | 7570538 | 76 |
| [SRR28623889](https://dataview.ncbi.nlm.nih.gov/object/SRR28623889) | [JBBFFX000000000](https://www.ncbi.nlm.nih.gov/nuccore/JBBFFX000000000) | SAMN40466909 | cjeju20_43 | 99.99x | 185286 | 7138718 | 233 |
| SRR28623878 | [JBBFFW000000000](https://www.ncbi.nlm.nih.gov/nuccore/JBBFFW000000000) | SAMN40466910 | cjeju20_50 | 99.98x | 153919 | 2833076 | 83 |
| [SRR28623867](https://dataview.ncbi.nlm.nih.gov/object/SRR28623867) | [JBBFFV000000000](https://www.ncbi.nlm.nih.gov/nuccore/JBBFFV000000000) | SAMN40466911 | cjeju20_51 | 99.98x | 160327 | 3445572 | 43 |
| [SRR28623856](https://dataview.ncbi.nlm.nih.gov/object/SRR28623856) | [JBBFFU000000000](https://www.ncbi.nlm.nih.gov/nuccore/JBBFFU000000000) | SAMN40466912 | cjeju20_53 | 99.99x | 128594 | 3418962 | 91 |
| [SRR28623845](https://dataview.ncbi.nlm.nih.gov/object/SRR28623845) | [JBBFFR000000000](https://www.ncbi.nlm.nih.gov/nuccore/JBBFFR000000000) | SAMN40466915 | cjeju20_57 | 99.99x | 153879 | 3531346 | 66 |
| [SRR28623834](https://dataview.ncbi.nlm.nih.gov/object/SRR28623834) | [JBBFFQ000000000](https://www.ncbi.nlm.nih.gov/nuccore/JBBFFQ000000000) | SAMN40466916 | cjeju20_59 | 99.99x | 140975 | 3024516 | 73 |
| [SRR28623823](https://dataview.ncbi.nlm.nih.gov/object/SRR28623823) | [JBBFFP000000000](https://www.ncbi.nlm.nih.gov/nuccore/JBBFFP000000000) | SAMN40466917 | cjeju20_60 | 99.98x | 190514 | 6592102 | 97 |
| [SRR28623921](https://dataview.ncbi.nlm.nih.gov/object/SRR28623921) | [JBBFFO000000000](https://www.ncbi.nlm.nih.gov/nuccore/JBBFFO000000000) | SAMN40466918 | cjeju20_61 | 99.98x | 194557 | 3283254 | 70 |
| [SRR28623910](https://dataview.ncbi.nlm.nih.gov/object/SRR28623910) | [JBBFFN000000000](https://www.ncbi.nlm.nih.gov/nuccore/JBBFFN000000000) | SAMN40466919 | cjeju20_62 | 99.98x | 142437 | 3363104 | 111 |
| [SRR28623908](https://dataview.ncbi.nlm.nih.gov/object/SRR28623908) | [JBBFFM000000000](https://www.ncbi.nlm.nih.gov/nuccore/JBBFFM000000000) | SAMN40466920 | cjeju20_8 | 99.98x | 153177 | 6740916 | 49 |
| [SRR28623907](https://dataview.ncbi.nlm.nih.gov/object/SRR28623907) | [JBBFFL000000000](https://www.ncbi.nlm.nih.gov/nuccore/JBBFFL000000000) | SAMN40466921 | cjeju21_1 | 99.99x | 153870 | 2188358 | 63 |
| [SRR28623906](https://dataview.ncbi.nlm.nih.gov/object/SRR28623906) | [JBBFFK000000000](https://www.ncbi.nlm.nih.gov/nuccore/JBBFFK000000000) | SAMN40466922 | cjeju21_13 | 99.99x | 253397 | 2411510 | 52 |
| [SRR28623905](https://dataview.ncbi.nlm.nih.gov/object/SRR28623905) | [JBBFFJ000000000](https://www.ncbi.nlm.nih.gov/nuccore/JBBFFJ000000000) | SAMN40466923 | cjeju21_14 | 99.98x | 141357 | 2507862 | 191 |
| [SRR28623904](https://dataview.ncbi.nlm.nih.gov/object/SRR28623904) | [JBBFFI000000000](https://www.ncbi.nlm.nih.gov/nuccore/JBBFFI000000000) | SAMN40466924 | cjeju21_15 | 99.99x | 187004 | 2652784 | 49 |
| [SRR28623903](https://dataview.ncbi.nlm.nih.gov/object/SRR28623903) | [JBBFFH000000000](https://www.ncbi.nlm.nih.gov/nuccore/JBBFFH000000000) | SAMN40466925 | cjeju21_16 | 99.99x | 117866 | 2309240 | 36 |
| [SRR28623902](https://dataview.ncbi.nlm.nih.gov/object/SRR28623902) | [JBBFFG000000000](https://www.ncbi.nlm.nih.gov/nuccore/JBBFFG000000000) | SAMN40466926 | cjeju21_17 | 99.99x | 116389 | 2421032 | 113 |
| [SRR28623901](https://dataview.ncbi.nlm.nih.gov/object/SRR28623901) | [JBBFFF000000000](https://www.ncbi.nlm.nih.gov/nuccore/JBBFFF000000000) | SAMN40466927 | cjeju21_18 | 99.99x | 159865 | 2317260 | 468 |
| [SRR28623899](https://dataview.ncbi.nlm.nih.gov/object/SRR28623899) | [JBBFFE000000000](https://www.ncbi.nlm.nih.gov/nuccore/JBBFFE000000000) | SAMN40466928 | cjeju21_19 | 99.98x | 178288 | 2514010 | 43 |
| [SRR28623898](https://dataview.ncbi.nlm.nih.gov/object/SRR28623898) | [JBBFFD000000000](https://www.ncbi.nlm.nih.gov/nuccore/JBBFFD000000000) | SAMN40466929 | cjeju21_2 | 99.99x | 234121 | 2520942 | 49 |
| [SRR28623897](https://dataview.ncbi.nlm.nih.gov/object/SRR28623897) | [JBBFFC000000000](https://www.ncbi.nlm.nih.gov/nuccore/JBBFFC000000000) | SAMN40466930 | cjeju21_21 | 100.0x | 190041 | 2177778 | 37 |
| [SRR28623896](https://dataview.ncbi.nlm.nih.gov/object/SRR28623896) | [JBBFFC000000000](https://www.ncbi.nlm.nih.gov/nuccore/JBBFFC000000000) | SAMN40466931 | cjeju21_22 | 99.99x | 221390 | 1783304 | 162 |
| [SRR28623895](https://dataview.ncbi.nlm.nih.gov/object/SRR28623895) | [JBBFFA000000000](https://www.ncbi.nlm.nih.gov/nuccore/JBBFFA000000000) | SAMN40466932 | cjeju21_23 | 99.97xx | 336543 | 2634846 | 202 |
| [SRR28623894](https://dataview.ncbi.nlm.nih.gov/object/SRR28623894) | [JBBFEZ000000000](https://www.ncbi.nlm.nih.gov/nuccore/JBBFEZ000000000) | SAMN40466933 | cjeju21_24 | 100.0x | 197767 | 2380728 | 34 |
| [SRR28623893](https://dataview.ncbi.nlm.nih.gov/object/SRR28623893) | [JBBFEX000000000](https://www.ncbi.nlm.nih.gov/nuccore/JBBFEX000000000) | SAMN40466934 | cjeju21_25 | 99.97x | 191030 | 1756604 | 54 |
| [SRR28623892](https://dataview.ncbi.nlm.nih.gov/object/SRR28623892) | [JBBFEX000000000](https://www.ncbi.nlm.nih.gov/nuccore/JBBFEX000000000) | SAMN40466935 | cjeju21_26 | 99.97x | 153872 | 2030236 | 52 |
| [SRR28623891](https://dataview.ncbi.nlm.nih.gov/object/SRR28623891) | [JBBFEW000000000](https://www.ncbi.nlm.nih.gov/nuccore/JBBFEW000000000) | SAMN40466936 | cjeju21_27 | 99.99x | 186576 | 2313994 | 212 |
| [SRR28623890](https://dataview.ncbi.nlm.nih.gov/object/SRR28623890) | [JBBFEV000000000](https://www.ncbi.nlm.nih.gov/nuccore/JBBFEV000000000) | SAMN40466937 | cjeju21_28 | 99.99x | 166365 | 2646550 | 75 |
| [SRR28623888](https://dataview.ncbi.nlm.nih.gov/object/SRR28623888) | [JBBFEU000000000](https://www.ncbi.nlm.nih.gov/nuccore/JBBFEU000000000) | SAMN40466938 | cjeju21_29 | 99.99x | 153872 | 1812620 | 55 |
| SRR28673397 | [JBBFET000000000](https://www.ncbi.nlm.nih.gov/nuccore/JBBFET000000000) | SAMN40466939 | cjeju21_31 | 99.98x | 179595 | 1790478 | 456 |
| [SRR28623887](https://dataview.ncbi.nlm.nih.gov/object/SRR28623887) | [JBBFES000000000](https://www.ncbi.nlm.nih.gov/nuccore/JBBFES000000000) | SAMN40466940 | cjeju21_32 | 99.98x | 161836 | 2048574 | 120 |
| [SRR28623886](https://dataview.ncbi.nlm.nih.gov/object/SRR28623886) | [JBBFER000000000](https://www.ncbi.nlm.nih.gov/nuccore/JBBFER000000000) | SAMN40466941 | cjeju21_33 | 99.98x | 176464 | 1659286 | 114 |
| [SRR28623885](https://dataview.ncbi.nlm.nih.gov/object/SRR28623885) | [JBBFEQ000000000](https://www.ncbi.nlm.nih.gov/nuccore/JBBFEQ000000000) | SAMN40466942 | cjeju21_34 | 99.99x | 153903 | 2164026 | 35 |
| [SRR28623884](https://dataview.ncbi.nlm.nih.gov/object/SRR28623884) | [JBBFEP000000000](https://www.ncbi.nlm.nih.gov/nuccore/JBBFEP000000000) | SAMN40466943 | cjeju21_35 | 99.98x | 154432 | 2109846 | 98 |
| [SRR28623883](https://dataview.ncbi.nlm.nih.gov/object/SRR28623883) | [JBBFEO000000000](https://www.ncbi.nlm.nih.gov/nuccore/JBBFEO000000000) | SAMN40466944 | cjeju21_36 | 99.99x | 135602 | 1941554 | 47 |
| [SRR28623882](https://dataview.ncbi.nlm.nih.gov/object/SRR28623882) | [JBBFEN000000000](https://www.ncbi.nlm.nih.gov/nuccore/JBBFEN000000000) | SAMN40466945 | cjeju21_37 | 99.99x | 155765 | 1962304 | 31 |
| [SRR28623881](https://dataview.ncbi.nlm.nih.gov/object/SRR28623881) | [JBBFEM000000000](https://www.ncbi.nlm.nih.gov/nuccore/JBBFEM000000000) | SAMN40466946 | cjeju21_8 | 99.98x | 161702 | 1996866 | 57 |
| [SRR28623880](https://dataview.ncbi.nlm.nih.gov/object/SRR28623880) | [JBBFEL000000000](https://www.ncbi.nlm.nih.gov/nuccore/JBBFEL000000000) | SAMN40466947 | cjeju22_10 | 99.99x | 188013 | 2273574 | 41 |
| [SRR28623879](https://dataview.ncbi.nlm.nih.gov/object/SRR28623879) | [JBBFEK000000000](https://www.ncbi.nlm.nih.gov/nuccore/JBBFEK000000000) | SAMN40466948 | cjeju22_100 | 99.99x | 184212 | 2394708 | 38 |
| [SRR28623877](https://dataview.ncbi.nlm.nih.gov/object/SRR28623877) | [JBBFEJ000000000](https://www.ncbi.nlm.nih.gov/nuccore/JBBFEJ000000000) | SAMN40466949 | cjeju22_101 | 99.99x | 153872 | 1995248 | 75 |
| [SRR28623876](https://dataview.ncbi.nlm.nih.gov/object/SRR28623876) | [JBBFEI000000000](https://www.ncbi.nlm.nih.gov/nuccore/JBBFEI000000000) | SAMN40466950 | cjeju22_103 | 99.99x | 213664 | 2113344 | 37 |
| [SRR28623875](https://dataview.ncbi.nlm.nih.gov/object/SRR28623875) | [JBBFEH000000000](https://www.ncbi.nlm.nih.gov/nuccore/JBBFEH000000000) | SAMN40466951 | cjeju22_106 | 99.98x | 195210 | 2081852 | 65 |
| [SRR28623874](https://dataview.ncbi.nlm.nih.gov/object/SRR28623874) | [JBBFEG000000000](https://www.ncbi.nlm.nih.gov/nuccore/JBBFEG000000000) | SAMN40466952 | cjeju22_108 | 99.98x | 185585 | 2405408 | 35 |
| [SRR28623873](https://dataview.ncbi.nlm.nih.gov/object/SRR28623873) | [JBBFEF000000000](https://www.ncbi.nlm.nih.gov/nuccore/JBBFEF000000000) | SAMN40466953 | cjeju22_109 | 99.98x | 157010 | 2582578 | 58 |
| [SRR28623872](https://dataview.ncbi.nlm.nih.gov/object/SRR28623872) | [JBBFEE000000000](https://www.ncbi.nlm.nih.gov/nuccore/JBBFEE000000000) | SAMN40466954 | cjeju22_11 | 99.99x | 129257 | 1937280 | 61 |
| [SRR28623871](https://dataview.ncbi.nlm.nih.gov/object/SRR28623871) | [JBBFED000000000](https://www.ncbi.nlm.nih.gov/nuccore/JBBFED000000000) | SAMN40466955 | cjeju22_110 | 99.99x | 153881 | 2182294 | 35 |
| [SRR28623870](https://dataview.ncbi.nlm.nih.gov/object/SRR28623870) | [JBBFEC000000000](https://www.ncbi.nlm.nih.gov/nuccore/JBBFEC000000000) | SAMN40466956 | cjeju22_111 | 100.0x | 153903 | 2265722 | 65 |
| [SRR28623869](https://dataview.ncbi.nlm.nih.gov/object/SRR28623869) | [JBBFEB000000000](https://www.ncbi.nlm.nih.gov/nuccore/JBBFEB000000000) | SAMN40466957 | cjeju22_112 | 99.99x | 168232 | 2256388 | 31 |
| [SRR28623868](https://dataview.ncbi.nlm.nih.gov/object/SRR28623868) | [JBBFEA000000000](https://www.ncbi.nlm.nih.gov/nuccore/JBBFEA000000000) | SAMN40466958 | cjeju22_114 | 100.0x | 153872 | 1826196 | 53 |
| [SRR28623866](https://dataview.ncbi.nlm.nih.gov/object/SRR28623866) | [JBBFDZ000000000](https://www.ncbi.nlm.nih.gov/nuccore/JBBFDZ000000000) | SAMN40466959 | cjeju22_115 | 99.97x | 220398 | 1786558 | 32 |
| [SRR28623865](https://dataview.ncbi.nlm.nih.gov/object/SRR28623865) | [JBBFDY000000000](https://www.ncbi.nlm.nih.gov/nuccore/JBBFDY000000000) | SAMN40466960 | cjeju22_116 | 99.99x | 334577 | 1877014 | 57 |
| [SRR28623864](https://dataview.ncbi.nlm.nih.gov/object/SRR28623864) | [JBBFDX000000000](https://www.ncbi.nlm.nih.gov/nuccore/JBBFDX000000000) | SAMN40466961 | cjeju22_12 | 99.99x | 113853 | 2382100 | 55 |
| [SRR28623863](https://dataview.ncbi.nlm.nih.gov/object/SRR28623863) | [JBBFDW000000000](https://www.ncbi.nlm.nih.gov/nuccore/JBBFDW000000000) | SAMN40466962 | cjeju22_125 | 100.0x | 186577 | 2156200 | 35 |
| [SRR28623862](https://dataview.ncbi.nlm.nih.gov/object/SRR28623862) | [JBBFDV000000000](https://www.ncbi.nlm.nih.gov/nuccore/JBBFDV000000000) | SAMN40466963 | cjeju22_126 | 100.0x | 106512 | 2175198 | 29 |
| [SRR28623861](https://dataview.ncbi.nlm.nih.gov/object/SRR28623861) | [JBBFDU000000000](https://www.ncbi.nlm.nih.gov/nuccore/JBBFDU000000000) | SAMN40466965 | cjeju22_16 | 99.99x | 153879 | 2166304 | 138 |
| [SRR28623860](https://dataview.ncbi.nlm.nih.gov/object/SRR28623860) | [JBBFDT000000000](https://www.ncbi.nlm.nih.gov/nuccore/JBBFDT000000000) | SAMN40466966 | cjeju22_18 | 99.98x | 298176 | 2071612 | 44 |
| [SRR28623859](https://dataview.ncbi.nlm.nih.gov/object/SRR28623859) | [JBBFDS000000000](https://www.ncbi.nlm.nih.gov/nuccore/JBBFDS000000000) | SAMN40466967 | cjeju22_19 | 99.98x | 188187 | 2714538 | 59 |
| [SRR28623858](https://dataview.ncbi.nlm.nih.gov/object/SRR28623858) | [JBBFDR000000000](https://www.ncbi.nlm.nih.gov/nuccore/JBBFDR000000000) | SAMN40466968 | cjeju22_2 | 99.99x | 177429 | 1723612 | 154 |
| [SRR28623857](https://dataview.ncbi.nlm.nih.gov/object/SRR28623857) | [JBBFDQ000000000](https://www.ncbi.nlm.nih.gov/nuccore/JBBFDQ000000000) | SAMN40466969 | cjeju22_20 | 99.99x | 120045 | 2232882 | 38 |
| [SRR28623855](https://dataview.ncbi.nlm.nih.gov/object/SRR28623855) | [JBBFDP000000000](https://www.ncbi.nlm.nih.gov/nuccore/JBBFDP000000000) | SAMN40466970 | cjeju22_21 | 100.0 | 184956 | 2091988 | 49 |
| [SRR28623854](https://dataview.ncbi.nlm.nih.gov/object/SRR28623854) | [JBBFDO000000000](https://www.ncbi.nlm.nih.gov/nuccore/JBBFDO000000000) | SAMN40466971 | cjeju22_32 | 99.99x | 183488 | 2985470 | 102 |
| [SRR28623853](https://dataview.ncbi.nlm.nih.gov/object/SRR28623853) | [JBBFDN000000000](https://www.ncbi.nlm.nih.gov/nuccore/JBBFDN000000000) | SAMN40466972 | cjeju22_33 | 99.99x | 220397 | 2601094 | 81 |
| [SRR28623852](https://dataview.ncbi.nlm.nih.gov/object/SRR28623852) | [JBBFDM000000000](https://www.ncbi.nlm.nih.gov/nuccore/JBBFDM000000000) | SAMN40466973 | cjeju22_4 | 99.99x | 160695 | 2050346 | 70 |
| [SRR28623851](https://dataview.ncbi.nlm.nih.gov/object/SRR28623851) | [JBBFDL000000000](https://www.ncbi.nlm.nih.gov/nuccore/JBBFDL000000000) | SAMN40466974 | cjeju22_48 | 99.98x | 179596 | 3012910 | 35 |
| [SRR28623850](https://dataview.ncbi.nlm.nih.gov/object/SRR28623850) | [JBBFDK000000000](https://www.ncbi.nlm.nih.gov/nuccore/JBBFDK000000000) | SAMN40466975 | cjeju22_49 | 99.99x | 178819 | 3234746 | 1601 |
| [SRR28623849](https://dataview.ncbi.nlm.nih.gov/object/SRR28623849) | [JBBFDJ000000000](https://www.ncbi.nlm.nih.gov/nuccore/JBBFDJ000000000) | SAMN40466976 | cjeju22_5 | 99.99x | 158338 | 2251096 | 105 |
| [SRR28623848](https://dataview.ncbi.nlm.nih.gov/object/SRR28623848) | [JBBFDI000000000](https://www.ncbi.nlm.nih.gov/nuccore/JBBFDI000000000) | SAMN40466977 | cjeju22_50 | 99.99x | 160665 | 2716196 | 34 |
| [SRR28623847](https://dataview.ncbi.nlm.nih.gov/object/SRR28623847) | [JBBFDH000000000](https://www.ncbi.nlm.nih.gov/nuccore/JBBFDH000000000) | SAMN40466978 | cjeju22_51 | 99.99x | 186576 | 2586870 | 29 |
| [SRR28623846](https://dataview.ncbi.nlm.nih.gov/object/SRR28623846) | [JBBFDG000000000](https://www.ncbi.nlm.nih.gov/nuccore/JBBFDG000000000) | SAMN40466979 | cjeju22_52 | 99.97x | 186595 | 3123780 | 58 |
| [SRR28623844](https://dataview.ncbi.nlm.nih.gov/object/SRR28623844) | [JBBFDF000000000](https://www.ncbi.nlm.nih.gov/nuccore/JBBFDF000000000) | SAMN40466980 | cjeju22_53 | 99.99x | 119380 | 2441372 | 35 |
| [SRR28623843](https://dataview.ncbi.nlm.nih.gov/object/SRR28623843) | [JBBFDE000000000](https://www.ncbi.nlm.nih.gov/nuccore/JBBFDE000000000) | SAMN40466981 | cjeju22_54 | 99.98x | 154154 | 3138134 | 77 |
| [SRR28623842](https://dataview.ncbi.nlm.nih.gov/object/SRR28623842) | [JBBFDD000000000](https://www.ncbi.nlm.nih.gov/nuccore/JBBFDD000000000) | SAMN40466982 | cjeju22_56 | 99.99x | 114939 | 2790972 | 33 |
| [SRR28623841](https://dataview.ncbi.nlm.nih.gov/object/SRR28623841) | [JBBFDC000000000](https://www.ncbi.nlm.nih.gov/nuccore/JBBFDC000000000) | SAMN40466983 | cjeju22_57 | 99.99x | 112646 | 2920886 | 38 |
| [SRR28623840](https://dataview.ncbi.nlm.nih.gov/object/SRR28623840) | [JBBFDB000000000](https://www.ncbi.nlm.nih.gov/nuccore/JBBFDB000000000) | SAMN40466984 | cjeju22_58 | 100.0x | 171801 | 2880842 | 32 |
| [SRR28623839](https://dataview.ncbi.nlm.nih.gov/object/SRR28623839) | [JBBFDA000000000](https://www.ncbi.nlm.nih.gov/nuccore/JBBFDA000000000) | SAMN40466985 | cjeju22_59 | 99.99x | 197766 | 2798634 | 64 |
| [SRR28623838](https://dataview.ncbi.nlm.nih.gov/object/SRR28623838) | [JBBFCZ000000000](https://www.ncbi.nlm.nih.gov/nuccore/JBBFCZ000000000) | SAMN40466986 | cjeju22_60 | 99.99x | 159521 | 2748228 | 125 |
| [SRR28623837](https://dataview.ncbi.nlm.nih.gov/object/SRR28623837) | [JBBFCY000000000](https://www.ncbi.nlm.nih.gov/nuccore/JBBFCY000000000) | SAMN40466987 | cjeju22_61 | 99.99x | 334479 | 3151850 | 65 |
| [SRR28623836](https://dataview.ncbi.nlm.nih.gov/object/SRR28623836) | [JBBFCX000000000](https://www.ncbi.nlm.nih.gov/nuccore/JBBFCX000000000) | SAMN40466988 | cjeju22_62 | 99.99x | 178121 | 2916998 | 59 |
| [SRR28623835](https://dataview.ncbi.nlm.nih.gov/object/SRR28623835) | [JBBFCW000000000](https://www.ncbi.nlm.nih.gov/nuccore/JBBFCW000000000) | SAMN40466989 | cjeju22_8 | 99.99x | 148383 | 2221748 | 201 |
| [SRR28623833](https://dataview.ncbi.nlm.nih.gov/object/SRR28623833) | [JBBFCV000000000](https://www.ncbi.nlm.nih.gov/nuccore/JBBFCV000000000) | SAMN40466990 | cjeju22_9 | 99.99x | 174741 | 2291652 | 43 |
| [SRR28623832](https://dataview.ncbi.nlm.nih.gov/object/SRR28623832) | [JBBFCU000000000](https://www.ncbi.nlm.nih.gov/nuccore/JBBFCU000000000) | SAMN40466991 | cjeju22_91 | 99.98x | 214759 | 1956940 | 83 |
| [SRR28623831](https://dataview.ncbi.nlm.nih.gov/object/SRR28623831) | [JBBFCT000000000](https://www.ncbi.nlm.nih.gov/nuccore/JBBFCT000000000) | SAMN40466992 | cjeju22_92 | 99.99x | 186577 | 1987012 | 32 |
| SRR28673396 | [JBBFCS000000000](https://www.ncbi.nlm.nih.gov/nuccore/JBBFCS000000000) | SAMN40466993 | cjeju22_93 | 99.99x | 152926 | 2181358 | 67 |
| [SRR28623830](https://dataview.ncbi.nlm.nih.gov/object/SRR28623830) | [JBBFCR000000000](https://www.ncbi.nlm.nih.gov/nuccore/JBBFCR000000000) | SAMN40466994 | cjeju22_98 | 100.0x | 160568 | 1794998 | 28 |
| [SRR28623829](https://dataview.ncbi.nlm.nih.gov/object/SRR28623829) | [JBBFCQ000000000](https://www.ncbi.nlm.nih.gov/nuccore/JBBFCQ000000000) | SAMN40466995 | cjeju22_99 | 99.98x | 106509 | 2498484 | 27 |
| [SRR28623828](https://dataview.ncbi.nlm.nih.gov/object/SRR28623828) | [JBBFCP000000000](https://www.ncbi.nlm.nih.gov/nuccore/JBBFCP000000000) | SAMN40466996 | cjeju23_101 | 99.99x | 159865 | 2234146 | 38 |
| [SRR28623827](https://dataview.ncbi.nlm.nih.gov/object/SRR28623827) | JBBFCO000000000 | SAMN40466997 | cjeju23_102 | 99.99x | 128167 | 2114884 | 51 |
| [SRR28623826](https://dataview.ncbi.nlm.nih.gov/object/SRR28623826) | [JBBFCN000000000](https://www.ncbi.nlm.nih.gov/nuccore/JBBFCN000000000) | SAMN40466998 | cjeju23_103 | 99.99x | 176182 | 2288428 | 109 |
| [SRR28623825](https://dataview.ncbi.nlm.nih.gov/object/SRR28623825) | [JBBFCM000000000](https://www.ncbi.nlm.nih.gov/nuccore/JBBFCM000000000) | SAMN40466999 | cjeju23_104 | 100.0x | 222823 | 2182680 | 31 |
| [SRR28623824](https://dataview.ncbi.nlm.nih.gov/object/SRR28623824) | [JBBFCL000000000](https://www.ncbi.nlm.nih.gov/nuccore/JBBFCL000000000) | SAMN40467000 | cjeju23_105 | 99.99x | 181325 | 2114520 | 70 |
| [SRR28623822](https://dataview.ncbi.nlm.nih.gov/object/SRR28623822) | [JBBFCK000000000](https://www.ncbi.nlm.nih.gov/nuccore/JBBFCK000000000) | SAMN40467001 | cjeju23_107 | 100.0x | 197775 | 2093676 | 40 |
| [SRR28623821](https://dataview.ncbi.nlm.nih.gov/object/SRR28623821) | [JBBFCJ000000000](https://www.ncbi.nlm.nih.gov/nuccore/JBBFCJ000000000) | SAMN40467002 | cjeju23_108 | 99.96x | 145879 | 2101796 | 28 |
| [SRR28623820](https://dataview.ncbi.nlm.nih.gov/object/SRR28623820) | [JBBFCI000000000](https://www.ncbi.nlm.nih.gov/nuccore/JBBFCI000000000) | SAMN40467003 | cjeju23_60 | 99.98x | 293640 | 2003508 | 37 |
| [SRR28623819](https://dataview.ncbi.nlm.nih.gov/object/SRR28623819) | [JBBFCH000000000](https://www.ncbi.nlm.nih.gov/nuccore/JBBFCH000000000) | SAMN40467004 | cjeju23_63 | 99.99x | 186577 | 2211508 | 36 |
| [SRR28623818](https://dataview.ncbi.nlm.nih.gov/object/SRR28623818) | [JBBFCG000000000](https://www.ncbi.nlm.nih.gov/nuccore/JBBFCG000000000) | SAMN40467005 | cjeju23_65 | 99.99x | 114939 | 2036700 | 32 |
| [SRR28623817](https://dataview.ncbi.nlm.nih.gov/object/SRR28623817) | [JBBFCF000000000](https://www.ncbi.nlm.nih.gov/nuccore/JBBFCF000000000) | SAMN40467006 | cjeju23_67 | 99.98x | 153995 | 2140738 | 40 |
| [SRR28623816](https://dataview.ncbi.nlm.nih.gov/object/SRR28623816) | [JBBFCE000000000](https://www.ncbi.nlm.nih.gov/nuccore/JBBFCE000000000) | SAMN40467007 | cjeju23_68 | 99.98x | 142433 | 1973948 | 46 |
| [SRR28623815](https://dataview.ncbi.nlm.nih.gov/object/SRR28623815) | [JBBFCD000000000](https://www.ncbi.nlm.nih.gov/nuccore/JBBFCD000000000) | SAMN40467008 | cjeju23_83 | 99.99x | 153911 | 2154656 | 23 |
| [SRR28623814](https://dataview.ncbi.nlm.nih.gov/object/SRR28623814) | [JBBFCC000000000](https://www.ncbi.nlm.nih.gov/nuccore/JBBFCC000000000) | SAMN40467009 | cjeju23_84 | 99.99x | 151685 | 2237872 | 32 |
| [SRR28623813](https://dataview.ncbi.nlm.nih.gov/object/SRR28623813) | [JBBFCB000000000](https://www.ncbi.nlm.nih.gov/nuccore/JBBFCB000000000) | SAMN40467010 | cjeju23_89 | 100.0x | 212985 | 2529646 | 29 |
| [SRR28623920](https://dataview.ncbi.nlm.nih.gov/object/SRR28623920) | [JBBFCA000000000](https://www.ncbi.nlm.nih.gov/nuccore/JBBFCA000000000) | SAMN40467011 | cjeju23_91 | 99.98x | 166153 | 1885540 | 59 |
| [SRR28623919](https://dataview.ncbi.nlm.nih.gov/object/SRR28623919) | [JBBFBZ000000000](https://www.ncbi.nlm.nih.gov/nuccore/JBBFBZ000000000) | SAMN40467012 | cjeju23_92 | 99.99x | 188160 | 2065332 | 64 |
| [SRR28623918](https://dataview.ncbi.nlm.nih.gov/object/SRR28623918) | [JBBFBY000000000](https://www.ncbi.nlm.nih.gov/nuccore/JBBFBY000000000) | SAMN40467013 | cjeju23_93 | 99.99x | 190186 | 3220464 | 60 |
| [SRR28623917](https://dataview.ncbi.nlm.nih.gov/object/SRR28623917) | [JBBFBX000000000](https://www.ncbi.nlm.nih.gov/nuccore/JBBFBX000000000) | SAMN40467014 | cjeju23_94 | 100.0x | 154048 | 2049780 | 80 |
| [SRR28623916](https://dataview.ncbi.nlm.nih.gov/object/SRR28623916) | [JBBFBW000000000](https://www.ncbi.nlm.nih.gov/nuccore/JBBFBW000000000) | SAMN40467015 | cjeju23_95 | 99.99x | 182518 | 2173654 | 32 |
| SRR28673396 | [JBBFBV000000000](https://www.ncbi.nlm.nih.gov/nuccore/JBBFBV000000000) | SAMN40467016 | cjeju24_1 | 99.98x | 186721 | 2758948 | 109 |
| [SRR28623915](https://dataview.ncbi.nlm.nih.gov/object/SRR28623915) | [JBBFBU000000000](https://www.ncbi.nlm.nih.gov/nuccore/JBBFBU000000000) | SAMN40467017 | cjeju24_10 | 99.98x | 187571 | 2178134 | 42 |
| [SRR28623914](https://dataview.ncbi.nlm.nih.gov/object/SRR28623914) | [JBBFBT000000000](https://www.ncbi.nlm.nih.gov/nuccore/JBBFBT000000000) | SAMN40467018 | cjeju24_3 | 99.99x | 161015 | 2407190 | 84 |
| [SRR28623913](https://dataview.ncbi.nlm.nih.gov/object/SRR28623913) | [JBBFBS000000000](https://www.ncbi.nlm.nih.gov/nuccore/JBBFBS000000000) | SAMN40467019 | cjeju24_5 | 99.99x | 190060 | 2330324 | 37 |
| [SRR28623912](https://dataview.ncbi.nlm.nih.gov/object/SRR28623912) | [JBBFBR000000000](https://www.ncbi.nlm.nih.gov/nuccore/JBBFBR000000000) | SAMN40467020 | cjeju24_6 | 99.99x | 153184 | 2389318 | 49 |
| [SRR28623911](https://dataview.ncbi.nlm.nih.gov/object/SRR28623911) | [JBBFBQ000000000](https://www.ncbi.nlm.nih.gov/nuccore/JBBFBQ000000000) | SAMN40467021 | cjeju24_8 | 99.99x | 186565 | 2233130 | 32 |
| [SRR28623909](https://dataview.ncbi.nlm.nih.gov/object/SRR28623909) | [JBBFBP000000000](https://www.ncbi.nlm.nih.gov/nuccore/JBBFBP000000000) | SAMN40467022 | cjeju24_9 | 99.99x | 186577 | 2432328 | 68 |

**Table** **S2.** Antimicrobial susceptibility, number of resistant antibiotic group, years of isolation and location of 114 *Campylobacter jejuni* isolates.

| **Isolate_ID** | **Nº of resistant antibiotic group** | **CIP** | **TET** | **E** | **Year of isolation** | **Location** |
| --- | --- | --- | --- | --- | --- | --- |
| cjeju20_3 | 2 | R | R | S | 2020 | Cadiz |
| cjeju20_4 | 2 | R | R | S | 2020 | San Fernando |
| cjeju20_42 | 2 | R | R | S | 2020 | Cadiz |
| cjeju20_43 | 2 | R | R | S | 2020 | San Fernando |
| cjeju20_50 | 2 | R | R | S | 2021 | Cadiz |
| cjeju20_51 | 2 | R | R | S | 2021 | San Fernando |
| cjeju20_53 | 2 | R | R | S | 2021 | Cadiz |
| cjeju20_57 | 2 | R | R | S | 2021 | San Fernando |
| cjeju20_59 | 2 | R | R | S | 2021 | Cadiz |
| cjeju20_60 | 1 | R | S | S | 2021 | Cadiz |
| cjeju20_61 | 1 | R | S | S | 2021 | Cadiz |
| cjeju20_62 | 2 | R | R | S | 2021 | Cadiz |
| cjeju20_8 | 2 | R | R | S | 2020 | San Fernando |
| cjeju21_1 | 0 | S | S | S | 2021 | Cadiz |
| cjeju21_13 | 2 | R | R | S | 2021 | Cadiz |
| cjeju21_14 | 2 | R | R | S | 2021 | Cadiz |
| cjeju21_15 | 2 | R | R | S | 2021 | Cadiz |
| cjeju21_16 | 1 | R | S | S | 2021 | Cadiz |
| cjeju21_17 | 0 | S | S | S | 2021 | Cadiz |
| cjeju21_18 | 2 | R | R | S | 2021 | Cadiz |
| cjeju21_19 | 1 | R | S | S | 2021 | Cadiz |
| cjeju21_2 | 1 | R | S | S | 2021 | Cadiz |
| cjeju21_21 | 2 | R | R | S | 2021 | San Fernando |
| cjeju21_22 | 1 | R | S | S | 2021 | Cadiz |
| cjeju21_23 | 2 | R | R | S | 2021 | San Fernando |
| cjeju21_24 | 2 | R | R | S | 2021 | San Fernando |
| cjeju21_25 | 2 | R | R | S | 2021 | San Fernando |
| cjeju21_26 | 2 | R | R | S | 2021 | Cadiz |
| cjeju21_27 | 2 | R | R | S | 2021 | Cadiz |
| cjeju21_28 | 2 | R | R | S | 2021 | Cadiz |
| cjeju21_29 | 1 | R | S | S | 2021 | Cadiz |
| cjeju21_31 | 2 | R | R | S | 2021 | Cadiz |
| cjeju21_32 | 2 | R | R | S | 2021 | Cadiz |
| cjeju21_33 | 2 | R | R | S | 2021 | Cadiz |
| cjeju21_34 | 1 | R | S | S | 2021 | Cadiz |
| cjeju21_35 | 1 | R | S | S | 2021 | Cadiz |
| cjeju21_36 | 2 | R | R | S | 2021 | Cadiz |
| cjeju21_37 | 0 | S | S | S | 2021 | San Fernando |
| cjeju21_8 | 2 | R | R | S | 2021 | Cadiz |
| cjeju22_10 | 1 | R | S | S | 2021 | Cadiz |
| cjeju22_100 | 0 | S | S | S | 2022 | Cadiz |
| cjeju22_101 | 1 | R | S | S | 2022 | Cadiz |
| cjeju22_103 | 1 | R | S | S | 2022 | Cadiz |
| cjeju22_106 | 2 | R | R | S | 2022 | Cadiz |
| cjeju22_108 | 2 | R | R | S | 2022 | Cadiz |
| cjeju22_109 | 2 | R | R | S | 2022 | Cadiz |
| cjeju22_11 | 2 | R | R | S | 2021 | Cadiz |
| cjeju22_110 | 1 | R | S | S | 2022 | Cadiz |
| cjeju22_111 | 1 | R | S | S | 2022 | Cadiz |
| cjeju22_112 | 2 | R | R | S | 2022 | Cadiz |
| cjeju22_114 | 2 | R | R | S | 2022 | Cadiz |
| cjeju22_115 | 2 | R | R | S | 2022 | Cadiz |
| cjeju22_116 | 0 | S | S | S | 2022 | Cadiz |
| cjeju22_12 | 2 | R | R | S | 2021 | Cadiz |
| cjeju22_125 | 2 | R | R | S | 2022 | San Fernando |
| cjeju22_126 | 2 | R | R | S | 2022 | San Fernando |
| cjeju22_16 | 1 | R | S | S | 2021 | Cadiz |
| cjeju22_18 | 2 | R | R | S | 2021 | Cadiz |
| cjeju22_19 | 2 | R | R | S | 2021 | Cadiz |
| cjeju22_2 | 1 | R | S | S | 2021 | Cadiz |
| cjeju22_20 | 2 | R | R | S | 2021 | Cadiz |
| cjeju22_21 | 2 | R | S | R | 2021 | Cadiz |
| cjeju22_32 | 0 | S | S | S | 2022 | Cadiz |
| cjeju22_33 | 1 | R | S | S | 2021 | Cadiz |
| cjeju22_4 | 2 | R | R | S | 2021 | Cadiz |
| cjeju22_48 | 0 | S | S | S | 2021 | Cadiz |
| cjeju22_49 | 2 | R | R | S | 2021 | Cadiz |
| cjeju22_5 | 2 | R | R | S | 2021 | Cadiz |
| cjeju22_50 | 1 | R | S | S | 2022 | Cadiz |
| cjeju22_51 | 2 | R | R | S | 2022 | Cadiz |
| cjeju22_52 | 2 | R | R | S | 2022 | Cadiz |
| cjeju22_53 | 1 | R | S | S | 2021 | Cadiz |
| cjeju22_54 | 2 | R | R | S | 2021 | Cadiz |
| cjeju22_56 | 2 | R | R | S | 2021 | San Fernando |
| cjeju22_57 | 2 | R | R | S | 2022 | Cadiz |
| cjeju22_58 | 2 | R | R | S | 2022 | Cadiz |
| cjeju22_59 | 2 | R | R | S | 2022 | Cadiz |
| cjeju22_60 | 1 | R | S | S | 2022 | Cadiz |
| cjeju22_61 | 2 | R | R | S | 2022 | Cadiz |
| cjeju22_62 | 1 | R | S | S | 2022 | San Fernando |
| cjeju22_8 | 1 | R | S | S | 2021 | Cadiz |
| cjeju22_9 | 2 | R | R | S | 2021 | Cadiz |
| cjeju22_91 | 1 | R | S | S | 2022 | Cadiz |
| cjeju22_92 | 2 | R | R | S | 2022 | Cadiz |
| cjeju22_93 | 0 | S | S | S | 2022 | Cadiz |
| cjeju22_98 | 0 | S | S | S | 2022 | Cadiz |
| cjeju22_99 | 2 | R | R | S | 2022 | Cadiz |
| cjeju23_101 | 2 | R | R | S | 2023 | Cadiz |
| cjeju23_102 | 1 | S | R | S | 2023 | Cadiz |
| cjeju23_103 | 2 | R | R | S | 2023 | San Fernando |
| cjeju23_104 | 2 | R | R | S | 2023 | San Fernando |
| cjeju23_105 | 2 | R | R | S | 2023 | San Fernando |
| cjeju23_107 | 2 | R | R | S | 2023 | San Fernando |
| cjeju23_108 | 2 | R | R | S | 2023 | Cadiz |
| cjeju23_60 | 2 | R | R | S | 2022 | Cadiz |
| cjeju23_63 | 2 | R | R | S | 2022 | Cadiz |
| cjeju23_65 | 1 | R | S | S | 2022 | San Fernando |
| cjeju23_67 | 1 | R | S | S | 2022 | Cadiz |
| cjeju23_68 | 1 | R | S | S | 2022 | Cadiz |
| cjeju23_83 | 2 | R | R | S | 2023 | Cadiz |
| cjeju23_84 | 2 | R | R | S | 2023 | Cadiz |
| cjeju23_89 | 2 | R | R | S | 2023 | Cadiz |
| cjeju23_91 | 1 | S | R | S | 2023 | Cadiz |
| cjeju23_92 | 2 | R | R | S | 2023 | Cadiz |
| cjeju23_93 | 2 | R | R | S | 2023 | Cadiz |
| cjeju23_94 | 1 | R | S | S | 2023 | Cadiz |
| cjeju23_95 | 2 | R | R | S | 2023 | Cadiz |
| cjeju24_1 | 2 | R | R | S | 2023 | Cadiz |
| cjeju24_10 | 2 | R | R | S | 2023 | San Fernando |
| cjeju24_3 | 2 | R | R | S | 2023 | Cadiz |
| cjeju24_5 | 2 | R | R | S | 2023 | Cadiz |
| cjeju24_6 | 1 | R | S | S | 2023 | Cadiz |
| cjeju24_8 | 2 | R | R | S | 2023 | Cadiz |
| cjeju24_9 | 2 | R | R | S | 2023 | San Fernando |

CIP: ciprofloxacin, E: erythromycin; TET: tetracycline

**Table** **S3.** Distribution of Clonal complex (CC), Sequnce Type (ST) and Core genome sequence type (cgMLST) on 114 *Campylobacter jejuni* isolates.

| **Isolate ID** | **Clonal complex (CC)** | **Sequence type (ST)** | **Core genome (cgMLST)** |
| --- | --- | --- | --- |
| cjeju20_3 | CC-42 complex | 6532 | 19242 |
| cjeju20_4 | CC-42 complex | 6532 | 35104 |
| cjeju20_42 | CC-42 complex | 6532 | 14959 |
| cjeju20_43 | CC-21 complex | 50 | 88265 |
| cjeju20_50 | CC-607 complex | 607 | 45535 |
| cjeju20_51 | CC-42 complex | 6532 | 14339, 19242 |
| cjeju20_53 | CC-354 complex | 354 | 48132 |
| cjeju20_57 | CC-21 complex | 19 | 48269 |
| cjeju20_59 | CC-443 complex | 6522 | 3286 |
| cjeju20_60 | CC-353 complex | 5 | 75401 |
| cjeju20_61 | CC-443 complex | 7517 | 20116 |
| cjeju20_62 | CC-42 complex | 6532 | 19242 |
| cjeju20_8 | CC-21 complex | 50 | 19078, 81895 |
| cjeju21_1 | CC-22 complex | 22 | 81330 |
| cjeju21_13 | CC-21 complex | 50 | 25897, 88265 |
| cjeju21_14 | CC-206 complex | 572 | 79973 |
| cjeju21_15 | CC-42 complex | 6532 | 19242 |
| cjeju21_16 | CC-443 complex | 7517 | 21997 |
| cjeju21_17 | CC-22 complex | 22 | 79379 |
| cjeju21_18 | CC-48 complex | 48 | 75718 |
| cjeju21_19 | CC-52 complex | 52 | 48512 |
| cjeju21_2 | CC-21 complex | 19 | 3520 |
| cjeju21_21 | CC-257 complex | 990 | 69637 |
| cjeju21_22 | CC-443 complex | 7517 | 21997 |
| cjeju21_23 | CC-353 complex | 400 | 35312, 45012 |
| cjeju21_24 | CC-21 complex | 50 | 3006 |
| cjeju21_25 | CC-206 complex | 122 | 48438 |
| cjeju21_26 | CC-21 complex | 50 | 18998, 81843 |
| cjeju21_27 | CC-574 complex | 3172 | 74312 |
| cjeju21_28 | CC-42 complex | 6532 | 19242 |
| cjeju21_29 | CC-354 complex | 9203 | 16975, 45360, 80470 |
| cjeju21_31 | CC-607 complex | 607 | 84646 |
| cjeju21_32 | CC-353 complex | 9887 | 20782 |
| cjeju21_33 | - | 531 | 101138 |
| cjeju21_34 | CC-353 complex | 2364 | 15041 |
| cjeju21_35 | CC-21 complex | 50 | 18726,81657 |
| cjeju21_36 | CC-48 complex | 48 | 75718 |
| cjeju21_37 | - | 1080 | 76297 |
| cjeju21_8 | CC-21 complex | 50 | 79730 |
| cjeju22_10 | CC-42 complex | 6532 | 35104 |
| cjeju22_100 | CC-45 complex | 583 | 71873 |
| cjeju22_101 | CC-353 complex | 5 | 75401 |
| cjeju22_103 | CC-206 complex | 122 | 3580 |
| cjeju22_106 | CC-257 complex | 824 | 80478 |
| cjeju22_108 | CC-206 complex | 572 | 85207 |
| cjeju22_109 | CC-21 complex | 982 | 18802 |
| cjeju22_11 | CC-21 complex | 50 | 18998, 81843 |
| cjeju22_110 | CC-21 complex | 19 | 70614 |
| cjeju22_111 | CC-443 complex | 7517 | 20116, 21997 |
| cjeju22_112 | CC-206 complex | 122 | 35432 |
| cjeju22_114 | CC-354 complex | 354 | 3662 |
| cjeju22_115 | CC-206 complex | 572 | 78144 |
| cjeju22_116 | CC-257 complex | 824 | 8646 |
| cjeju22_12 | CC-574 complex | 3172 | 74312 |
| cjeju22_125 | CC-42 complex | 6532 | 14339 |
| cjeju22_126 | CC-206 complex | 572 | 78144 |
| cjeju22_16 | CC-354 complex | 9203 | 80470 |
| cjeju22_18 | CC-354 complex | 354 | 3662 |
| cjeju22_19 | CC-21 complex | 21 | 21464 |
| cjeju22_2 | CC-21 complex | 19 | 3520 |
| cjeju22_20 | Unassigend | 2133 | 72177 |
| cjeju22_21 | CC-42 complex | 6532 | 35104 |
| cjeju22_32 | CC-61 complex | 61 | 102335 |
| cjeju22_33 | CC-353 complex | 353 | 68510 |
| cjeju22_4 | CC-206 complex | 572 | 79973 |
| cjeju22_48 | CC-45 complex | 583 | 68071 |
| cjeju22_49 | CC-353 complex | 7355 | 34814, 35324 |
| cjeju22_5 | CC-21 complex | 21 | 580 |
| cjeju22_50 | CC-52 complex | 52 | 48512 |
| cjeju22_51 | CC-206 complex | 572 | 85207 |
| cjeju22_52 | CC-21 complex | 50 | 101149 |
| cjeju22_53 | CC-42 complex | 6532 | 35488 |
| cjeju22_54 | CC-257 complex | 824 | 80478 |
| cjeju22_56 | CC-353 complex | 356 | 13832 |
| cjeju22_57 | Unassigend | 2133 | 72177 |
| cjeju22_58 | CC-206 complex | 572 | 78144 |
| cjeju22_59 | CC-354 complex | 354 | 3662 |
| cjeju22_60 | CC-21 complex | 148 | 19245 |
| cjeju22_61 | CC-206 complex | 572 | 101146 |
| cjeju22_62 | CC-22 complex | 22 | 70553 |
| cjeju22_8 | CC-21 complex | 19 | 3520 |
| cjeju22_9 | CC-42 complex | 6532 | 35104 |
| cjeju22_91 | CC-353 complex | 6461 | 4628 |
| cjeju22_92 | CC-353 complex | 356 | 13832 |
| cjeju22_93 | CC-179 complex | 4447 | 99837, 99838 |
| cjeju22_98 | Unassigend | 2861 | 78144, 18045, 18046, 18047, 50989, 50990, 50991, 81273, 81274, 81275 |
| cjeju22_99 | CC-206 complex | 572 | 78144 |
| cjeju23_101 | CC-206 complex | 572 | 85207 |
| cjeju23_102 | CC-21 complex | 19 | 19368 |
| cjeju23_103 | CC-353 complex | 6461 | 4628 |
| cjeju23_104 | CC-48 complex | 48 | 77195 |
| cjeju23_105 | CC-607 complex | 607 | 4193 |
| cjeju23_107 | CC-206 complex | 122 | 35260 |
| cjeju23_108 | CC-206 complex | 572 | 85207 |
| cjeju23_60 | CC-42 complex | 517 | 35488 |
| cjeju23_63 | CC-48 complex | 48 | 75718 |
| cjeju23_65 | CC-52 complex | 52 | 48465 |
| cjeju23_67 | CC-443 complex | 7517 | 21997 |
| cjeju23_68 | CC-443 complex | 51 | 19911 |
| cjeju23_83 | CC-257 complex | 2254 | 18653 |
| cjeju23_84 | CC-257 complex | 2254 | 18653 |
| cjeju23_89 | CC-21 complex | 50 | 3006 |
| cjeju23_91 | CC-21 complex | 19 | 19368 |
| cjeju23_92 | CC-353 complex | 1232 | 20645 |
| cjeju23_93 | CC-353 complex | 8334 | 35609 |
| cjeju23_94 | CC-21 complex | 861 | 19143 |
| cjeju23_95 | CC-354 complex | 354 | 47011 |
| cjeju24_1 | CC-21 complex | 148 | 19245 |
| cjeju24_10 | CC-22 complex | 22 | 100524 |
| cjeju24_3 | CC-353 complex | 9390 | 45948 |
| cjeju24_5 | CC-206 complex | 572 | 101146 |
| cjeju24_6 | CC-45 complex | 418 | 73957 |
| cjeju24_8 | CC-206 complex | 572 | 101146 |
| cjeju24_9 | CC-354 complex | 354 | 5005 |

**Table** **S4** Phenotypic and genotype profiles of 114 *Campylobacter jejuni* isolates.

| **Isolate ID** | **Genotype CIP** | **Phenotype CIP** | **Genotype TET** | **Phenotype TET** | **Genotype E** | **Phenotype E** |
| --- | --- | --- | --- | --- | --- | --- |
| cjeju20_3 | gyrA p.T86I | R | *tet(0)* | R |  | S |
| cjeju20_4 | gyrA p.T86I | R | *tet(0)* | R |  | S |
| cjeju20_42 | gyrA p.T86I | R | *tet(0)* | R |  | S |
| cjeju20_43 | gyrA p.T86I | R | *tet(0)* | R |  | S |
| cjeju20_50 | gyrA p.T86I | R | *tet(0)* | R |  | S |
| cjeju20_51 | gyrA p.T86I | R | *tet(0)* | R |  | S |
| cjeju20_53 | gyrA p.T86I | R | *tet(0)* | R |  | S |
| cjeju20_57 | gyrA p.T86I | R | *tet(0)* | R |  | S |
| cjeju20_59 | gyrA p.T86I | R | *tet(0)* | R |  | S |
| cjeju20_60 | gyrA p.T86I | R |  | S |  | S |
| cjeju20_61 | gyrA p.T86I | R |  | S |  | S |
| cjeju20_62 | gyrA p.T86I | R | *tet(0)* | R |  | S |
| cjeju20_8 | gyrA p.T86I | R | *tet(0)* | R |  | S |
| cjeju21_1 |  | S |  | S |  | S |
| cjeju21_13 | gyrA p.T86I | R | *tet(0)* | R |  | S |
| cjeju21_14 | gyrA p.T86I | R | *tet(0)* | R |  | S |
| cjeju21_15 | gyrA p.T86I | R | *tet(0)* | R |  | S |
| cjeju21_16 | gyrA p.T86I | R |  | S |  | S |
| cjeju21_17 |  | S |  | S |  | S |
| cjeju21_18 | gyrA p.T86I | R | *tet(0)* | R |  | S |
| cjeju21_19 | gyrA p.T86I | R |  | S |  | S |
| cjeju21_2 | gyrA p.T86I | R |  | S |  | S |
| cjeju21_21 | gyrA p.T86I | R | *tet(0)* | R |  | S |
| cjeju21_22 | gyrA p.T86I | R |  | S |  | S |
| cjeju21_23 | gyrA p.T86I | R | *tet(0)* | R |  | S |
| cjeju21_24 | gyrA p.T86I | R | *tet(0)* | R |  | S |
| cjeju21_25 | gyrA p.T86I | R | *tet(0)* | R |  | S |
| cjeju21_26 | gyrA p.T86I | R | *tet(0)* | R |  | S |
| cjeju21_27 | gyrA p.T86I | R | *tet(0)* | R |  | S |
| cjeju21_28 | gyrA p.T86I | R | *tet(0)* | R |  | S |
| cjeju21_29 | gyrA p.T86I | R |  | S |  | S |
| cjeju21_31 | gyrA p.T86I | R | *tet(0)* | R |  | S |
| cjeju21_32 | gyrA p.T86I | R | *tet(0)* | R |  | S |
| cjeju21_33 | gyrA p.T86I | R | *tet(0)* | R |  | S |
| cjeju21_34 | gyrA p.T86I | R |  | S |  | S |
| cjeju21_35 | gyrA p.T86I | R | *tet(0)* | S |  | S |
| cjeju21_36 | gyrA p.T86I | R | *tet(0)* | R |  | S |
| cjeju21_37 |  | S |  | S |  | S |
| cjeju21_8 | gyrA p.T86I | R | *tet(0)* | R |  | S |
| cjeju22_10 | gyrA p.T86I | R |  | S |  | S |
| cjeju22_100 |  | S |  | S |  | S |
| cjeju22_101 | gyrA p.T86I | R |  | S |  | S |
| cjeju22_103 | gyrA p.T86I | R |  | S |  | S |
| cjeju22_106 | gyrA p.T86I | R | *tet(0)* | R |  | S |
| cjeju22_108 | gyrA p.T86I | R | *tet(0)* | R |  | S |
| cjeju22_109 | gyrA p.T86I | R | *tet(0)* | R |  | S |
| cjeju22_11 | gyrA p.T86I | R | *tet(0)* | R |  | S |
| cjeju22_110 | gyrA p.T86I | R |  | S |  | S |
| cjeju22_111 | gyrA p.T86I | R |  | S |  | S |
| cjeju22_112 | gyrA p.T86I | R | *tet(0)* | R |  | S |
| cjeju22_114 | gyrA p.T86I | R | *tet(0)* | R |  | S |
| cjeju22_115 | gyrA p.T86I | R | *tet(0)* | R |  | S |
| cjeju22_116 |  | S |  | S |  | S |
| cjeju22_12 | gyrA p.T86I | R | *tet(0)* | R |  | S |
| cjeju22_125 | gyrA p.T86I | R | *tet(0)* | R |  | S |
| cjeju22_126 | gyrA p.T86I | R | *tet(0)* | R |  | S |
| cjeju22_16 | gyrA p.T86I | R |  | S |  | S |
| cjeju22_18 | gyrA p.T86I | R | *tet(0)* | R |  | S |
| cjeju22_19 | gyrA p.T86I | R | *tet(0)* | R |  | S |
| cjeju22_2 | gyrA p.T86I | R |  | S |  | S |
| cjeju22_20 | gyrA p.T86I | R | *tet(0)* | R |  | S |
| cjeju22_21 | gyrA p.T86I | R |  | S |  | R |
| cjeju22_32 |  | S |  | S |  | S |
| cjeju22_33 | gyrA p.T86I | R |  | S |  | S |
| cjeju22_4 | gyrA p.T86I | R | *tet(0)* | R |  | S |
| cjeju22_48 |  | S |  | S |  | S |
| cjeju22_49 | gyrA p.T86I | R | *tet(0)* | R |  | S |
| cjeju22_5 | gyrA p.T86I | R | *tet(0)* | R |  | S |
| cjeju22_50 |  | R |  | S |  | S |
| cjeju22_51 | gyrA p.T86I | R | *tet(0)* | R |  | S |
| cjeju22_52 | gyrA p.T86I | R | *tet(0)* | R |  | S |
| cjeju22_53 | gyrA p.T86I | R |  | S |  | S |
| cjeju22_54 | gyrA p.T86I | R | *tet(0)* | R |  | S |
| cjeju22_56 | gyrA p.T86I | R | *tet(0)* | R |  | S |
| cjeju22_57 | gyrA p.T86I | R | *tet(0)* | R |  | S |
| cjeju22_58 | gyrA p.T86I | R | *tet(0)* | R |  | S |
| cjeju22_59 | gyrA p.D90N; gyrA p.T86I | R | *tet(0)* | R |  | S |
| cjeju22_60 | gyrA p.T86I | R |  | S |  | S |
| cjeju22_61 | gyrA p.T86I | R | *tet(0)* | R |  | S |
| cjeju22_62 | gyrA p.T86I | R |  | S |  | S |
| cjeju22_8 | gyrA p.T86I | R |  | S |  | S |
| cjeju22_9 | gyrA p.T86I | R | *tet(0)* | R |  | S |
| cjeju22_91 | gyrA p.T86I | R |  | S |  | S |
| cjeju22_92 | gyrA p.T86I | R | *tet(0)* | R |  | S |
| cjeju22_93 |  | S |  | S |  | S |
| cjeju22_98 |  | S |  | S |  | S |
| cjeju22_99 | gyrA p.T86I | R | *tet(0)* | R |  | S |
| cjeju23_101 | gyrA p.T86I | R | *tet(0)* | R |  | S |
| cjeju23_102 |  | S | *tet(0)* | R |  | S |
| cjeju23_103 | gyrA p.T86I | R | *tet(0)* | R |  | S |
| cjeju23_104 | gyrA p.T86I | R | *tet(0)* | R |  | S |
| cjeju23_105 | gyrA p.T86I | R | *tet(0)* | R |  | S |
| cjeju23_107 | gyrA p.T86I | R | *tet(0)* | R |  | S |
| cjeju23_108 | gyrA p.T86I | R | *tet(0)* | R |  | S |
| cjeju23_60 | gyrA p.T86I | R | *tet(0)* | R |  | S |
| cjeju23_63 | gyrA p.T86I | R | *tet(0)* | R |  | S |
| cjeju23_65 | gyrA p.T86I | R |  | S |  | S |
| cjeju23_67 | gyrA p.T86I | R |  | S |  | S |
| cjeju23_68 | gyrA p.T86I | R |  | S |  | S |
| cjeju23_83 | gyrA p.T86I | R | *tet(0)* | R |  | S |
| cjeju23_84 | gyrA p.T86I | R | *tet(0)* | R |  | S |
| cjeju23_89 | gyrA p.T86I | R | *tet(0)* | R |  | S |
| cjeju23_91 |  | S | *tet(0)* | R |  | S |
| cjeju23_92 | gyrA p.T86I | R | *tet(0)* | R |  | S |
| cjeju23_93 | gyrA p.T86I | R | *tet(0)* | R |  | S |
| cjeju23_94 | gyrA p.T86I | R |  | S |  | S |
| cjeju23_95 | gyrA p.T86I | R | *tet(0)* | R |  | S |
| cjeju24_1 | gyrA p.T86I | R |  | R |  | S |
| cjeju24_10 | gyrA p.T86I | R | *tet(0)* | R |  | S |
| cjeju24_3 | gyrA p.T86I | R | *tet(0)* | R |  | S |
| cjeju24_5 | gyrA p.T86I | R | *tet(0)* | R |  | S |
| cjeju24_6 |  | R |  | S |  | S |
| cjeju24_8 | gyrA p.T86I | R | *tet(0)* | R |  | S |
| cjeju24_9 | gyrA p.T86I | R | *tet(0)* | R |  | S |

CIP: ciprofloxacin, E: erythromycin; TET: tetracycline

**Table S5. Detection of RE-cmeABC variant in the 114 C. jejuni isolates.**

| **Isolate ID** | **Ciprofloxacin MIC (mg/L)** | **Identity blastn**  **RE-cmeABC (%) *** | **Identity blastn**  **CmeB (%) ^*`*^** | **CmeABC operon** |
| --- | --- | --- | --- | --- |
| cjeju20_3 | - | 86 | 98,88 | cmeA,cmeB, cmeC |
| cjeju20_4 | - | 86,08 | 98,88 | cmeA,cmeB, cmeC |
| cjeju20_42 | - | 86 | 98,88 | cmeA,cmeB, cmeC |
| cjeju20_43 | - | 86 | 100 | cmeA,cmeB, cmeC |
| cjeju20_50 | >32 | 95 | 78,75 | cmeA,cmeC |
| cjeju20_51 | - | 86,08 | 98,88 | cmeA,cmeB, cmeC |
| cjeju20_53 | - | 86,32 | 98,59 | cmeA,cmeB, cmeC |
| cjeju20_57 | - | 86,38 | 100 | cmeA,cmeB, cmeC |
| cjeju20_59 | - | 86,35 | 96,82 | cmeA,cmeB, cmeC |
| cjeju20_60 | - | 86,24 | 98,66 | cmeA,cmeB, cmeC |
| cjeju20_61 | - | 86,19 | 98,53 | cmeA,cmeB, cmeC |
| cjeju20_62 | - | 86,08 | 98,88 | cmeA,cmeB, cmeC |
| cjeju20_8 | - | 86,38 | 100 | cmeA,cmeB, cmeC |
| cjeju21_1 | - | 86,24 | 98,85 | cmeA,cmeB, cmeC |
| cjeju21_13 | - | 86,24 | 100 | cmeA,cmeB, cmeC |
| cjeju21_14 | - | 86,25 | 98,69 | cmeA,cmeB, cmeC |
| cjeju21_15 | - | 86,08 | 98,88 | cmeA,cmeB, cmeC |
| cjeju21_16 | - | 86,19 | 98,53 | cmeA,cmeB, cmeC |
| cjeju21_17 | - | 86,24 | 98,85 | cmeA,cmeB, cmeC |
| cjeju21_18 | - | 86,1 | 99,94 | cmeA,cmeB, cmeC |
| cjeju21_19 | - | 86,21 | 98,01 | cmeA,cmeB, cmeC |
| cjeju21_2 | - | 86,37 | 99,94 | cmeA,cmeB, cmeC |
| cjeju21_21 | >32 | 95 | 79,42 | cmeA,cmeC |
| cjeju21_22 | - | 86,19 | 98,53 | cmeA,cmeB, cmeC |
| cjeju21_23 | - | 86,25 | 98,69 | cmeA,cmeB, cmeC |
| cjeju21_24 | - | 86,37 | 100 | cmeA,cmeB, cmeC |
| cjeju21_25 | - | 86,35 | 99,97 | cmeA,cmeB, cmeC |
| cjeju21_26 | - | 86,38 | 100 | cmeA,cmeB, cmeC |
| cjeju21_27 | >32 | 94,71 | 79,61 | cmeA,cmeC |
| cjeju21_28 | - | 86,08 | 98,88 | cmeA,cmeB, cmeC |
| cjeju21_29 | - | 86,32 | 98,56 | cmeA,cmeB, cmeC |
| cjeju21_31 | >32 | 95,5 | 78,75 | cmeA,cmeC |
| cjeju21_32 | - | 86,25 | 98,69 | cmeA,cmeB, cmeC |
| cjeju21_33 | >32 | 95,3 | 79,09 | cmeA,cmeC |
| cjeju21_34 | - | 86,24 | 98,11 | cmeA,cmeB, cmeC |
| cjeju21_35 | - | 86,38 | 100 | cmeA,cmeB, cmeC |
| cjeju21_36 | - | 86,1 | 99,94 | cmeA,cmeB, cmeC |
| cjeju21_37 | 0,125 | 94,57 | 78,72 | cmeA,cmeC |
| cjeju21_8 | - | 86,38 | 100 | cmeA,cmeB, cmeC |
| cjeju22_10 | - | 86,08 | 98,88 | cmeA,cmeB, cmeC |
| cjeju22_100 | - | 86,29 | 98,85 | cmeA,cmeB, cmeC |
| cjeju22_101 | - | 86,24 | 98,66 | cmeA,cmeB, cmeC |
| cjeju22_103 | - | 86,35 | 99,97 | cmeA,cmeB, cmeC |
| cjeju22_106 | - | 86,29 | 98,53 | cmeA,cmeB, cmeC |
| cjeju22_108 | - | 86,38 | 99,97 | cmeA,cmeB, cmeC |
| cjeju22_109 | - | 86,35 | 99,97 | cmeA,cmeB, cmeC |
| cjeju22_11 | - | 86,38 | 100 | cmeA,cmeB, cmeC |
| cjeju22_110 | - | 86,26 | 99,94 | cmeA,cmeB, cmeC |
| cjeju22_111 | - | 86,19 | 98,53 | cmeA,cmeB, cmeC |
| cjeju22_112 | - | 86,35 | 99,97 | cmeA,cmeB, cmeC |
| cjeju22_114 | - | 86,32 | 98,59 | cmeA,cmeB, cmeC |
| cjeju22_115 | - | 86,35 | 100 | cmeA,cmeB, cmeC |
| cjeju22_116 | - | 86,29 | 98,53 | cmeA,cmeB, cmeC |
| cjeju22_12 | - | 86,19 | 97,98 | cmeA,cmeB, cmeC |
| cjeju22_125 | - | 86,08 | 98,88 | cmeA,cmeB, cmeC |
| cjeju22_126 | - | 86,35 | 100 | cmeA,cmeB, cmeC |
| cjeju22_16 | - | 86,32 | 98,56 | cmeA,cmeB, cmeC |
| cjeju22_18 | - | 86,32 | 98,59 | cmeA,cmeB, cmeC |
| cjeju22_19 | - | 86,34 | 99,97 | cmeA,cmeB, cmeC |
| cjeju22_2 | - | 86,37 | 99,94 | cmeA,cmeB, cmeC |
| cjeju22_20 | - | 86,3 | 98,66 | cmeA,cmeB, cmeC |
| cjeju22_21 | - | 86,08 | 98,88 | cmeA,cmeB, cmeC |
| cjeju22_32 | - | 86,32 | 99,97 | cmeA,cmeB, cmeC |
| cjeju22_33 | - | 86,25 | 98,69 | cmeA,cmeB, cmeC |
| cjeju22_4 | - | 86,25 | 98,69 | cmeA,cmeB, cmeC |
| cjeju22_48 | - | 86,29 | 98,85 | cmeA,cmeB, cmeC |
| cjeju22_49 | - | 86,29 | 99,46 | cmeA,cmeB, cmeC |
| cjeju22_5 | - | 95,5 | 79,8 | cmeA,cmeC |
| cjeju22_50 | - | 86,21 | 98,01 | cmeA,cmeB, cmeC |
| cjeju22_51 | - | 86,38 | 99,97 | cmeA,cmeB, cmeC |
| cjeju22_52 | - | 86,08 | 100 | cmeA,cmeB, cmeC |
| cjeju22_53 | - | 86,08 | 98,88 | cmeA,cmeB, cmeC |
| cjeju22_54 | - | 86,29 | 98,53 | cmeA,cmeB, cmeC |
| cjeju22_56 | - | 86,24 | 98,11 | cmeA,cmeB, cmeC |
| cjeju22_57 | - | 86,3 | 98,66 | cmeA,cmeB, cmeC |
| cjeju22_58 | - | 86,35 | 100 | cmeA,cmeB, cmeC |
| cjeju22_59 | - | 86,32 | 98,59 | cmeA,cmeB, cmeC |
| cjeju22_60 | - | 86,37 | 99,97 | cmeA,cmeB, cmeC |
| cjeju22_61 | - | 86,25 | 98,69 | cmeA,cmeB, cmeC |
| cjeju22_62 | - | 86,24 | 98,72 | cmeA,cmeB, cmeC |
| cjeju22_8 | - | 86,37 | 99,94 | cmeA,cmeB, cmeC |
| cjeju22_9 | - | 86,08 | 98,88 | cmeA,cmeB, cmeC |
| cjeju22_91 | - | 86,27 | 98,69 | cmeA,cmeB, cmeC |
| cjeju22_92 | - | 86,24 | 98,11 | cmeA,cmeB, cmeC |
| cjeju22_93 | - | 86,38 | 97,92 | cmeA,cmeB, cmeC |
| cjeju22_98 | - | 86,13 | 98,56 | cmeA,cmeB, cmeC |
| cjeju22_99 | - | 86,35 | 100 | cmeA,cmeB, cmeC |
| cjeju23_101 | - | 86,25 | 99,97 | cmeA,cmeB, cmeC |
| cjeju23_102 | - | 86,38 | 100 | cmeA,cmeB, cmeC |
| cjeju23_103 | - | 86,27 | 98,69 | cmeA,cmeB, cmeC |
| cjeju23_104 | - | 86,1 | 99,94 | cmeA,cmeB, cmeC |
| cjeju23_105 | >32 | 95,37 | 78,75 | cmeA,cmeC |
| cjeju23_107 | - | 86,37 | 99,94 | cmeA,cmeB, cmeC |
| cjeju23_108 | - | 86,38 | 99,97 | cmeA,cmeB, cmeC |
| cjeju23_60 | - | 86,08 | 98,88 | cmeA,cmeB, cmeC |
| cjeju23_63 | - | 86,1 | 99,94 | cmeA,cmeB, cmeC |
| cjeju23_65 | - | 86,21 | 98,01 | cmeA,cmeB, cmeC |
| cjeju23_67 | - | 86,19 | 98,53 | cmeA,cmeB, cmeC |
| cjeju23_68 | - | 86,19 | 98,53 | cmeA,cmeB, cmeC |
| cjeju23_83 | - | 86,33 | 98,78 | cmeA,cmeB, cmeC |
| cjeju23_84 | - | 86,33 | 98,78 | cmeA,cmeB, cmeC |
| cjeju23_89 | - | 86,38 | 100 | cmeA,cmeB, cmeC |
| cjeju23_91 | - | 86,38 | 100 | cmeA,cmeB, cmeC |
| cjeju23_92 | - | 86,21 | 97,92 | cmeA,cmeB, cmeC |
| cjeju23_93 | - | 86,25 | 98,69 | cmeA,cmeB, cmeC |
| cjeju23_94 | - | 86,35 | 98,69 | cmeA,cmeB, cmeC |
| cjeju23_95 | - | 86,32 | 98,59 | cmeA,cmeB, cmeC |
| cjeju24_1 | - | 86,37 | 99,97 | cmeA,cmeB, cmeC |
| cjeju24_10 | - | 86,21 | 98,78 | cmeA,cmeB, cmeC |
| cjeju24_3 | >32 | 95,63 | 78,78 | cmeA,cmeC |
| cjeju24_5 | - | 86,25 | 98,69 | cmeA,cmeB, cmeC |
| cjeju24_6 | - | 86,37 | 97,89 | cmeA,cmeB, cmeC |
| cjeju24_8 | - | 86,25 | 98,69 | cmeA,cmeB, cmeC |
| cjeju24_9 | - | 86,32 | 98,59 | cmeA,cmeB, cmeC |

^*^  GenBank genome accession: KT778507.1

^**^ GenBank genome accession: NC_002163.1:c335718-332596
